# Andrographolide Inhibits Epstein–Barr Virus Lytic Reactivation in EBV-Positive Cancer Cell Lines through the Modulation of Epigenetic-Related Proteins

**DOI:** 10.3390/molecules27144666

**Published:** 2022-07-21

**Authors:** Praphatson Malat, Tipaya Ekalaksananan, Chukkris Heawchaiyaphum, Supawadee Suebsasana, Sittiruk Roytrakul, Yodying Yingchutrakul, Chamsai Pientong

**Affiliations:** 1 Department of Microbiology, Faculty of Medicine, Khon Kaen University, Khon Kaen 40002, Thailand; phatson88@gmail.com (P.M.); tipeka@kku.ac.th (T.E.); 2 HPV & EBV and Carcinogenesis Research Group, Khon Kaen University, Khon Kaen 40002, Thailand; 3 Department of Biotechnology, Faculty of Science and Technology, Rangsit Center, Thammasart University, Pathum Thani 12120, Thailand; jukkris.003@gmail.com; 4 Department of Pharmaceutical Sciences, Faculty of Pharmacy, Thammasat University, Bangkok 10200, Thailand; hnungnet@yahoo.com; 5 Proteomics Research Laboratory, Genome Technology Research Unit, National Center for Genetic Engineering and Biotechnology, National Science and Technology Development Agency, Pathum Thani 12120, Thailand; sittiruk@biotec.or.th (S.R.); yodying.yin@nstda.or.th (Y.Y.)

**Keywords:** andrographolide, Epstein–Barr virus, lytic reactivation, BZLF1, histone modifications

## Abstract

Reactivation of Epstein–Barr virus (EBV) is associated with EBV-associated malignancies and is considered to be a benefit target for treatment. Andrographolide is claimed to have antiviral and anti-tumor activities. Therefore, this study aimed to investigate the effect of andrographolide on the inhibition of EBV lytic reactivation in EBV-positive cancer cells. The cytotoxicity of andrographolide was firstly evaluated in EBV-positive cancer cells; P3HR1, AGS-EBV and HONE1-EBV cells, using an MTT assay. Herein, the spontaneous expression of EBV lytic genes; BALF5, BRLF1 and BZLF1, was significantly inhibited in andrographolide-treated cells. Accordingly, andrographolide inhibited the expression of Zta and viral production in sodium butyrate (NaB)-induced EBV lytic reactivation. Additionally, proteomics and bioinformatics analysis revealed the differentially expressed proteins that inhibit EBV lytic reactivation in all treated cell lines were functionally related with the histone modifications and chromatin organization, such as histone H3-K9 modification and histone H3-K27 methylation. Taken together, andrographolide inhibits EBV reactivation in EBV-positive cancer cells by inhibiting EBV lytic genes, probably, through the histone modifications.

## 1. Introduction

Epstein–Barr virus (EBV) is an important human pathogen with a worldwide distribution. Most people in developing countries acquire the infection in the first few years of life, often by ages 3–4 years, whereas infection in developed countries is often delayed until adolescence [1,2]. About 95% of the worldwide population has persistent EBV latent infection. EBV is a causative agent of several human diseases and has been associated with numerous types of human cancer including gastric cancer (GC), Burkett’s lymphoma (BL) and nasopharyngeal carcinoma (NPC), post-transplant lymphoproliferative diseases (PTLDs) and Hodgkin’s lymphoma [3]. The primary route of EBV transmission is oral, between parents and infants, siblings, friends in school or colleges, or adults in the household. In addition, it has been reported that organ transplantation and blood transfusion can also lead to EBV spread [4]. In the human host, EBV has two distinct phases. In “latent infection”, the virus persists in a dormant state for the lifetime of the host. In the lytic phase, the virus produces new infectious virions. The switch from latency to lytic infection is known as EBV reactivation. In cell-culture systems, cells with latent EBV infection can be stimulated to enter the lytic phase by chemical induction or biological agents such as NaB, 12-O-tetradecanoyl-phorbol-1,3-acetate (TPA), anti-immunoglobulin G (IgG) antibodies and some cytokines. Upon reactivation, EBV expresses sequentially lytic protein; immediate early (IE), early (E) and late (L) proteins, to produce the mature virions that are transmitted from cell to cell and between hosts [5,6].

Although it is thought that EBV latency participates in carcinogenesis, increasing evidence has suggested that EBV reactivation plays an important role in the pathogenesis of several human cancers. Wu, et al. [7]. found that high EBV DNA viral load and antibody titers against EBV lytic proteins are associated with a high risk of NPC. In addition, recurrent EBV reactivation is a necessary process to promote invasiveness, tumorigenesis, genome instability, angiogenesis and metastasis [8,9].

Andrographolide is extracted from *Andrographis paniculata* (Burm. f.) Nees (family *Acanthaceae*), and is widely founded in Asia [10,11,12]. This compound exhibits pharmacological activities including anti-inflammatory, immunomodulatory, anti-oxidant, anti-microbial and anti-viral effects [13,14]. Previous studies have indicated the therapeutic role of andrographolide in several human diseases [15,16].

However, the association between biological functions and antiviral effects of andrographolide on EBV-infected cancer cells is less well understood. We hypothesize that blocking EBV reactivation using andrographolide delays the progression of tumorigenesis and the inhibition of EBV reactivation prevents the production of EBV, which might reduce the contribution of EBV infection of neighboring cells. Therefore, this study aimed to investigate the ability of andrographolide to inhibit EBV reactivation and its association with common protein profiles in EBV-associated cancer cell lines.

## 2. Results

### 2.1. Cytotoxicity of Andrographolide

A colorimetric of 3-(4,5-dimethylthiazole-2-yl)-2,5-diphenyltetrazolium bromide (MTT) assay was performed to assess the cytotoxicity of andrographolide in EBV-positive cell lines; a Burkitt’s lymphoma cell line (P3HR1), and epithelial cells including a nasopharyngeal carcinoma (NPC) cell line (HONE1-EBV) and a gastric adenocarcinoma (GC) cell line (AGS-EBV). 

The dose-dependent reduction in cell viability was observed in all cells that were treated with andrographolide. Accordingly, the concentration μM) of andrographolide required for the reduction in cell viability by the 50% cytotoxic concentration (CC_50_) as well as 25% cytotoxic concentration (CC_25_) and 15% cytotoxic concentration (CC_15_) were calculated. The sub-toxic concentrations of andrographolide at CC_15_ and CC_25_ (Table 1) for each cell line were used for further experiments. 

### 2.2. Andrographolide Inhibit the Spontaneous Lytic Reactivation of EBV

**Figure 1:**
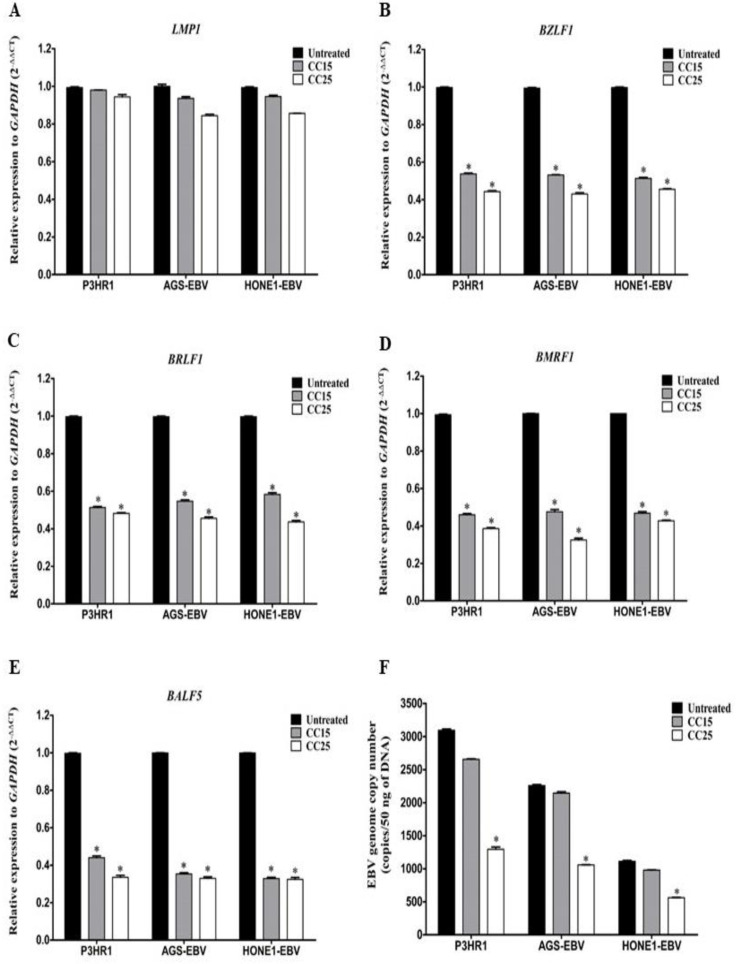
Andrographolide suppresses the spontaneous expression of EBV lytic genes. Cells were treated with various concentrations of andrographolide for 48 h. The expression of LMP1 (**A**), BZLF1 (**B**), BRLF1 (**C**), BMRF1 (**D**) and BALF5 (**E**) genes were analyzed by qRT-PCR. The quantification of EBV genome copy number (**F**) was performed by qPCR. *: *p* < 0.05.

To examine whether andrographolide inhibits the spontaneous lytic reactivation of EBV, EBV-positive cells were either treated with or without andrographolide at CC_15_ and CC_25_ for 48 h. We found that the expression of the EBV latent gene, LMP1 was not significantly different between treated and untreated cells (Figure 1A). On the contrary, the expression levels of EBV lytic genes, including BZLF1, BMRF1, BRLF1 and BALF5 were significantly decreased in andrographolide-treated cells (Figure 1B–E), suggesting andrographolide is a suppressor of spontaneous lytic gene expression. Consistent with this finding, the EBV genome copy numbers in supernatant from andrographolide-treated cells were significantly decreased when compared with untreated cells (Figure 1F). Therefore, these results suggest that andrographolide inhibits the spontaneous lytic reactivation of EBV infection in EBV-positive cancer cells.

### 2.3. Andrographolide Treatment and EBV Lytic Gene Induction

**Figure 2:**
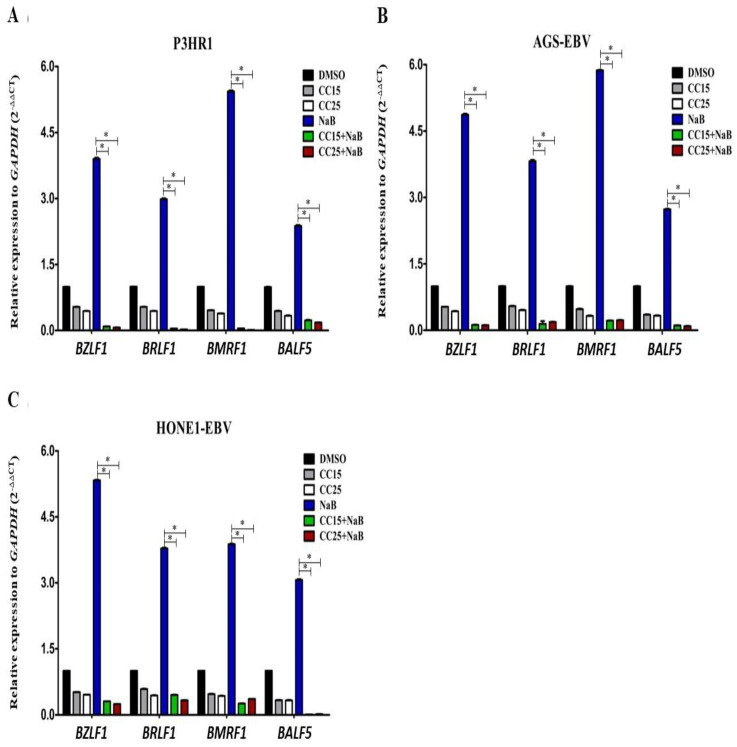
Andrographolide inhibits the induction of EBV lytic reactivation. Cells were treated with and without andrographolide at CC_15_ and CC_25_ for 3 h, and NaB was subsequently added, and further incubated for 48 h. The expression levels of BZLF1, BRLF1, BMRF1 and BALF5 genes in P3HR1 (**A**), AGS-EBV (**B**) and HONE1-EBV (**C**) cells were examined using qRT-PCR. *: *p* < 0.05.

To confirm whether andrographolide suppresses EBV lytic reactivation, cells were treated with andrographolide for 3 h and subsequently induced the lytic replication of EBV by NaB treatment and further incubated for 48 h. As shown in Figure 2, the expression of EBV lytic genes was highly expressed in NaB-treated cells. On the other hand, the expression of EBV lytic genes was dramatically decreased in cells treated with a combination of andrographolide (CC_15_ and CC_25_) and NaB when compared with NaB alone (Figure 2). Thus, this result suggests that andrographolide, at sub-toxic concentrations, inhibits EBV lytic reactivation by, at least, inhibiting the expression of lytic genes.

**Figure 3:**
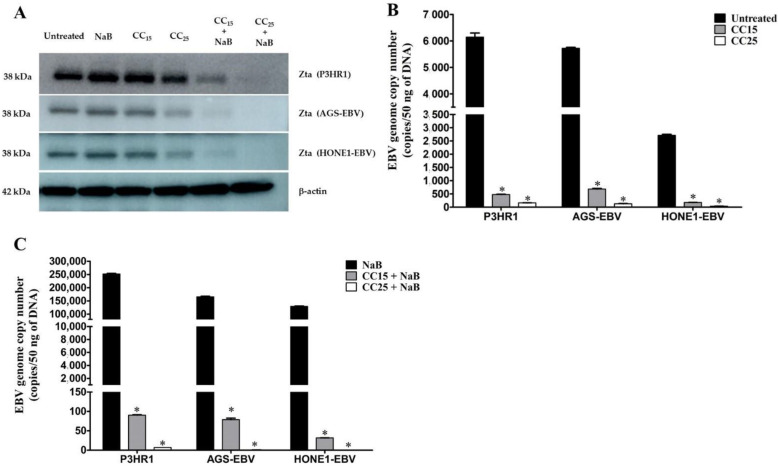
Andrographolide suppresses Zta expression and inhibits EBV copy number. Cells were treated with andrographolide for 3 h, subsequently treated with NaB and further incubated for 48 h. (**A**) The expression of Zta protein in cell lines was analyzed by western blotting. EBV genome copy number was quantified by qPCR. Cells were either treated with or without andrographolide at CC_15_ and CC_25_ (**B**) and cells were treated with a combination of andrographolide and NaB (**C**). The copy number of the EBV genome was calculated using a linear regression equation. *: *p* < 0.05.

Since we found that andrographolide inhibited the expression of EBV lytic genes under the induction of EBV lytic replication, we further confirmed the result by determining the expression of EBV immediate-early protein, Zta, in cells that were either treated with andrographolide, or a combination of andrographolide and NaB. Consistent with the mRNA level, the expression of Zta was obviously decreased in cells treated with andrographolide. Interestingly, the expression of Zta was completely inhibited in the cell treated with a combination of andrographolide and NaB (Figure 3A). More detail about Zta expression is shown in Figures S1 and S2. Collectively, these results demonstrate that andrographolide suppresses EBV lytic reactivation by inhibiting EBV lytic genes at both mRNA and protein levels.

Furthermore, to quantify the EBV virion production, the EBV copy number was determined by qPCR, cells were pre-treated with andrographolide at CC_15_ and CC_25_ for 3 h, and subsequently incubated with or without NaB for 48 h. As expected, andrographolide significantly inhibited the EBV copy number in a dose-dependent manner (Figure 3B). In addition, the EBV copy number was almost completely inhibited in cells that were treated with a combination of andrographolide and NaB (Figure 3C). These results indicate that andrographolide inhibited EBV virion production.

### 2.4. Andrographolide Induces Histone Modification-Related Proteins in EBV-Positive Cancer Cell Lines

**Figure 4:**
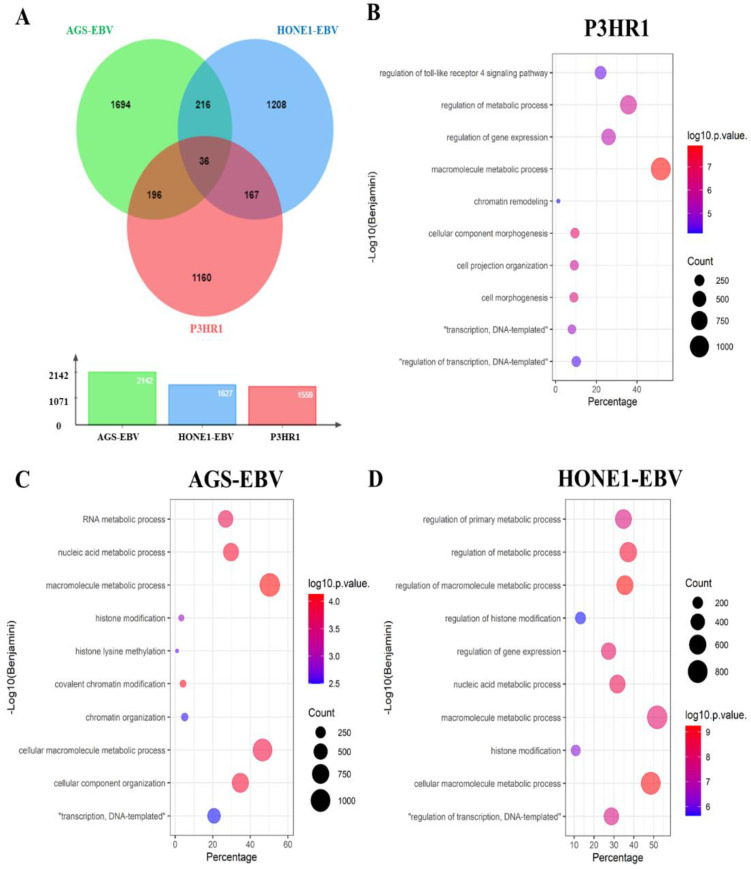
Venn diagram analysis of differential expressed proteins among EBV-positive cell lines (P3HR1, AGS-EBV and HONE1-EBV). Cells were either treated with DMSO (control), NaB or the combination of andrographolide and NaB. The total proteins were extracted and subjected to LC-MS/MS for proteomic analysis. The differentially expressed proteins from 3 different cell lines individually. All expressed proteins in cells that were NaB or the combination of andrographolide and NaB were firstly normalized with cells that were treated with DMSO. Then, the differentially expressed in cells that were treated with the combination of andrographolide and NaB were analyzed by comparing their expression levels with the proteins expressed in cells that were treated with NaB. The extracted proteins were subject to LC-MS/MS, then protein data were analyzed by MaxQuant. (**A**) The total identified proteins were analyzed using Venn diagram analysis. Andrographolide treatment alters host gene expression in EBV-infected cells and inhibits EBV lytic reactivation by induction of histone modifications. The differential expressed proteins were functionally analyzed in the context of biological processes in P3HR1 (**B**), AGS-EBV (**C**) and HONE1-EBV (**D**) by PANTHER.

Our previous study demonstrated that andrographolide inhibits EBV lytic reactivation by inhibition of transcription factors (TFs) expression in EBV-positive gastric cancer cell line (AGS-EBV) [17]. Therefore, we further investigated the molecular mechanism by which andrographolide inhibits EBV lytic reactivation in three different cell lines, including P3HR1, AGS-EBV and HONE1-EBV cells, using proteomics and bioinformatics analysis. The proteins that were differentially expressed in cells treated with andrographolide and induced the lytic reactivation of EBV by NaB were analyzed for their functionals using PANTHER. As expected, andrographolide modulated the expression of proteins. We found that 2142, 1627 and 1559 proteins were differentially expressed in andrographolide and NaB-treated AGS-EBV, HONE1-EBV and P3HR1, respectively (Figure 4A). The top 100 up-regulated and down-regulated proteins (raw data) in cells treated with andrographolide and a combination of andrographolide and NaB (Table S1). In addition, we found that 36 proteins were commonly up-regulated in all cell lines that were treated with a combination of andrographolide and NaB (Table S2). These differentially expressed proteins were involved in various biological processes. Of these, the common biological processes were the regulation of gene expression, chromatin remodeling histone modification and covalent chromatin modification (Figure 4D).

**Figure 5:**
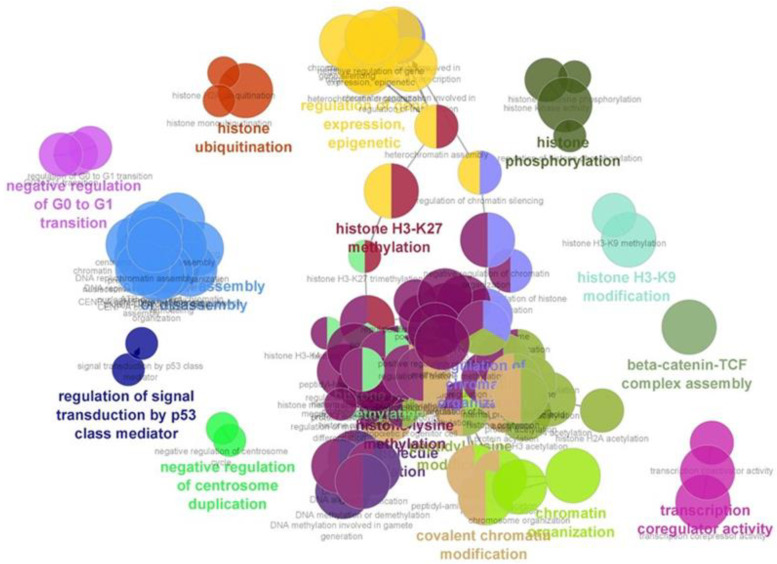
Andrographolide inhibits EBV lytic reactivation by induction of histone modifications. The differentially expressed proteins that were associated with histone modifications were analyzed for their molecular function by ClueGO. These proteins were functionally associated with histone H3-K27 methylation, histone H3-K9 modification, histone ubiquitination, β-catenin-TCF complex assembly, regulation of signal transduction by p53 class mediator, negative regulation of G0 to G1 transition, covalent chromatin modification and others.

Accumulating evidence demonstrated that the histone modifications can inhibit the lytic reactivation of EBV through the silencing of EBV lytic gene expression [18,19]. In addition, we also found that histone modification-associated proteins were differentially expressed in a combination of andrographolide and NAB-treated cells. Therefore, we further investigated the underlying mechanism of these differentially expressed proteins that may play important roles in the inhibition of EBV reactivation by bioinformatics analysis using ClueGO. As shown in Figure 5, the proteins that were differentially expressed in all cells treated with a combination of andrographolide and NaB were associated with the histone modifications, such as histone H3-K27 methylation, histone H3-K9 modification, histone ubiquitination, β-catenin-TCF complex assembly, regulation of signal transduction by p53 class mediator, negative regulation of G0 to G1 transition, covalent chromatin modification and others (Table S1). In addition, to better understand, we have added the data of biological processes that associate with the differentially expressed proteins, as in Table S3. Therefore, these results suggest that andrographolide inhibits EBV lytic reactivation in EBV-positive cell lines by the induction of histone modifications and chromatin organization. Taken together, our findings demonstrate that andrographolide inhibits lytic reactivation of EBV by the silencing of lytic genes through histone modifications.

## 3. Discussion

EBV lytic infection likely plays a role in the development and progression of cancers and transmission of EBV [20,21] Therefore, we hypothesized that inhibition of lytic reactivation would inhibit the transmission of EBV that may suppress EBV-induced moncogenesis. Here, we show that andrographolide can inhibit lytic reactivation by inhibiting the expression of EBV lytic genes at both mRNA and protein levels.

It is well demonstrated that latent infection of EBV is tightly associated with the pathogenesis of EBV-induced malignancies. However, the lytic infection phase of EBV is considered to play a role in carcinogenesis. Thus, the development of an effective strategy to inhibit the lytic cycle of EBV may be valuable in reducing the transmission of the virus and risk of EBV-associated diseases.

Accumulating evidence demonstrated that the lytic reactivation of EBV can promote the development and progression of EBV-associated cancers [21,22]. By enhancing transmission of the virus from cell to cell, EBV lytic infection can increase the total number of infected cells, a key factor of viral pathogenesis. The recurrence of EBV reactivation might induce genome instability and chromosomal aberration in NPC cells, thus enhancing tumorigenicity. In addition, the induction of EBV lytic reactivation could promote the invasiveness and tumorigenicity of NPC cells [23], suggesting its potential role in promoting tumor growth and progression. Therefore, the inhibition of EBV reactivation could be a vital process for the treatment of EBV-associated diseases.

The natural compounds that were extracted and purified from plants are known to inhibit EBV infection. For example, glycyrrhizic acid, derived from licorice root, can inhibit the replication of EBV by interfering with viral attachment and penetration [5]. The (-)-epigallocatechin gallate (EGCG) extracted from green tea and moronic acid extracted from Polygonum cuspidatum inhibit the expression of Rta, Zta and EAD proteins of EBV [24,25]. In addition, resveratrol, a non-flavonoid polyphenol compound extracted from various plants and fruits, inhibits the expression of the immediate-early genes BZLF1 and BRLF1 genes and EA-D protein and also reduces virion production [26].

In the present study, we demonstrated that andrographolide, a compound extracted from *Andrographis paniculata*, significantly suppressed EBV lytic reactivation by inhibiting the expression of EBV lytic genes and viral copy number in both lymphoma cell (P3HR1) and epithelial cells (AGS-EBV and HONE1-EBV). It was consistent with a previous study, which demonstrated that andrographolide inhibits EBV lytic reactivation in P3HR1 cells by inhibiting the expression of Zta, Rta and EA-D proteins and also inhibition of the promoter activity of Zp and Rp [27]. In addition, the derivative of andrographolide, 14-α-lipoylandrographolide (AL-1), exhibited a potent agent against human influenza viruses, H1N1 and H9N2, both in vitro and in vitro [28]. The infectivity of HIV was also suppressed by a derivative of andrographolide by inhibiting gp120-mediated cell fusion of HL2/3 cells with TZM-bl cells [29]. In addition, andrographolide can also suppress cancer progression by induction of cytotoxicity, cell cycle arrest, apoptosis and autophagy [30,31,32,33]. The treatment of NPC cells with andrographolide could inhibit the proliferation and invasiveness of NPC cells. In addition, andrographolide could also induce apoptosis, cell death and cell-cycle arrest via the NF-κB pathway [33].

The dysregulation of epigenetic machineries can interrupt the normal expression of tumor suppressor genes (TSGs) and oncogenes, which may ultimately lead to tumorigenesis [34]. Previous study has demonstrated that EBV lytic reactivation was inhibited by histone modification through the function of histone H3 lysine 27 trimethylation (H3K27me3) and histone H4 Lysine 20 (H4K20me3), which can silence the expression of BZLF1 gene in Raji cells [18,19]. The expression of EZH2, which encodes a histone H3K27 methyltransferase, plays an important role in inhibiting EBV lytic replication and progeny production in Akata-EBV cells [35]. Consistently, we also found that proteins that were associated with the histone modifications, such as histone H3-K27 methylation, histone H3-K9 modification, histone ubiquitination, α-catenin-TCF complex assembly, regulation of signal transduction by p53 class mediator, negative regulation of G0 to G1 transition, and covalent chromatin modification, were significantly enriched in cells treated with andrographolide and NaB. Therefore, these results suggest that andrographolide inhibits EBV lytic reactivation in EBV-positive cell lines by induction of histone modifications and chromatin organization. An andrographolide might be a promising therapeutic compound against EBV-associated malignancies.

## 4. Materials and Methods

### 4.1. Cell Lines and Andrographolide

The EBV-positive cells: P3HR1, HONE1-EBV and AGS-EBV cell lines were kindly provided by Prof. Hironori Yoshiyama (Shimane University, Japan). These cells were cultured in RPMI 1640 medium containing 10% FBS and antibiotics (streptomycin and penicillin). The andrographolide compound was prepared as previously described [36].

### 4.2. Cytotoxicity Assay

The cytotoxicity of andrographolide was evaluated using an MTT assay (Sigma-Aldrich, Saint Louis, MI, USA). Briefly, P3HR1 cells (5 × 10^5^ cells/mL), AGS-EBV and HONE1-EBV cells (1 × 10^5^ cells/mL) were seeded in 96-well plates and incubated for 24 h. Cells were treated with various concentrations of andrographolide (12.5, 25, 50 and 100 μM) and further incubated for 24 h. The 5 mg/mL of MTT was added and the crystals were dissolved by adding 200 μL of dimethyl sulfoxide (DMSO). The MTT signal was measured at 540 nm using a microplate reader. The results were reported as CC_50_, CC_25_ and CC_15._

### 4.3. Andrographolide Treatment and EBV Lytic Gene Induction

Cells were either treated with or without andrographolide at CC_15_ and CC_25_ for 3 h and subsequently induced by NaB for EBV lytic reactivation. All cells were further incubated for 48 h to examine whether andrographolide is an inducer or a suppressor of EBV lytic reactivation. Total RNA was extracted from the treated cells using TRIzol™ reagent (Invitrogen, Waltham, MA, USA) according to the manufacturer’s instructions. cDNA was synthesized using RevertAid First Strand cDNA Synthesis Kit (Thermo Scientific, Waltham, MA, USA) according to the manufacturer’s instructions. The quantitative expression of EBV genes was determined using qRT-PCR using SsoAdvancedTM SYBR® Green Supermix (BIO-RAD, Hercules, CA, USA) and was performed in triplicate on an Applied Biosystems QuantStudio6 instrument (Thermo Scientific, MA, USA). The expression of EBV genes was evaluated using the comparative CT method and normalized using GAPDH as the endogenous control. The sequences of primers used in this study are listed in Table 2. Relative expression levels of EBV genes were examined by the 2^−ΔΔCT^ method.

### 4.4. Determination of EBV Lytic Protein

To confirm whether andrographolide suppresses EBV lytic reactivation, the EBV lytic protein, Zta, was assayed in all cell lines treated with andrographolide by Western blotting. Total proteins (50 μg protein) were resolved by sodium dodecyl sulfate-polyacrylamide gel electrophoresis (SDS-PAGE) and transferred to a nitrocellulose membrane (Merck KGaA, Darmstadt, Germany) The membrane was incubated with primary antibody (mouse monoclonal antibody; anti-BZLF1 antibody (Santa Cruz Biotechnology, Dallas, TX, USA); anti-β-actin (Abcam, Cambridge, UK) and further incubated with secondary antibody (goat anti-mouse IgG-HRP, ECMTM, UK)). For chemiluminescence detection, the membrane was incubated with a substrate mixture (Millipore, ImmobilonTM Western chemiluminescent HRP substrate, Burlington, MA, USA). The signal was detected by Image Quant LAS 4000 mini version 1.1 (GE Healthcare, Upsala, Sweden). The expression of β-actin was used as a reference.

### 4.5. Quantification of EBV Genome Copy Number

The pGEM-T vector containing EBNA1 was transformed into a competent cell (*E. coli*, DH5α). Bacteria carrying the plasmid were grown and extracted for the plasmids using E.Z.N.A.^®^ Plasmid DNA Mini Kit I (E.Z.N.A.^®^ Plasmid Mini Kit I, Omega Bio-tek^®^, Norcross, GA, USA) according to the manufacturer’s instructions. The concentration of plasmid was quantified by NanoDrop. The presence of EBNA1 was detected by PCR using EBNA1 primer pair (Table 2) and constructed the standard linear regression equation.

To determine whether andrographolide inhibits EBV virion production, EBV copy number was determined in the supernatant collected from cells that were either treated with CC_15_ or CC_25_ of andrographolide or NaB alone or a combination of CC_15_ or CC_25_ of andrographolide and NaB for 48 h. The supernatant was harvested by centrifugation and further extracted for DNA by adding cell lysis solution according to the manufacturer’s instructions (QIAGEN, Redwood City, CA, USA). The copy number of EBV genomes was estimated from the standard curve of EBV copy number by using CT values obtained from samples and the standard linear regression equation. The results were expressed by means of copies/50 ng of DNA derived from three independent experiments.

### 4.6. Proteomics and Bioinformatic Analysis

Cells that were either treated with andrographolide or NaB or DMSO (control) for 48 h. In addition, we also pre-treated with andrographolide for 3 h and subsequently induced the lytic reactivation of EBV by NaB and further incubated for 48 h. The total proteins were extracted and evaluated for the protein content by the Lowry method. To investigate the expression profiles of proteins, total protein (10 μg) was firstly packed with 12.5% poly acrylamide and in-gel digestion was performed as described previously [17]. The tryptic peptide samples were injected into an Ultimate 3000 Nano/Capillary LC System (Thermo Scientific, Waltham, MA, USA) coupled to a Hybrid quadrupole Q-Tof impact II™ (Bruker, Billerica, MA, USA) equipped with a nano-captive spray ion source. The MaxQuant version 1.6.1.12 was used to quantify the proteins in individual samples using the Andromeda search engine to correlate MS/MS spectra to the Uniprot database. To identify the differentially expressed proteins, the jvenn web tool (http://jvenn.toulouse.inra.fr, accessed on 5 November 2021) was used [40]. The functional annotation of the unique proteins was performed by PANTHER and ClueGO [41].

### 4.7. Statistical Analysis

GraphPad Prism was used for all data analysis. One-way analysis of variance (ANOVA) and Mann-Whitney tests were used to test whether there was a difference between two independent groups expressed as mean ± standard deviation (SD). All experiments were repeated three times. All *p*-values less than 0.05 were considered statistically significant.

## 5. Conclusions

Andrographolide inhibits the lytic reactivation of EBV by suppressing the expression of EBV lytic genes and the production of EBV virion, probably via epigenetic modifications, particularly histone modifications. Therefore, andrographolide might be a promising therapeutic compound against EBV-associated malignancies

## Figures and Tables

**Table 1:** The CC_90_, CC_50_, CC_25_ and CC_15_ values for andrographolide were evaluated in P3HR1, AGS-EBV and HONE1-EBV cells.

EBV-Positive Cells	Andrographolide Concentration (μM)			
	CC_90_	CC_50_	CC_25_	CC_15_
P3HR1	116.51	69.80	38.70	26.25
AGS-EBV	108.20	67.72	34.40	21.07
HONE1-EBV	115.64	69.41	36.33	23.10

**Table 2:** EBV primer pairs [37,38,39].

EBV Genes	Primer Sequence (5′-3′)	Product Size (bp)
GAPDH	F: TCATCAGCAATGCCTCCTGCA R: TGGGTGGCAGTGATGGCA	117
BZLF1	F: TGTTTCAACCGCTCCGACTG R: GGGTTATGTCGGAGACTGGG	110
BRLF1	F: TGTTTCAACCGCTCCGACTG R: GGGTTATGTCGGAGACTGGG	94
BMRF1	F: ACCTGCCGTTGGATCTTAGTG R: GGCGTTGTTGGAGTCCTGTG	129
BALF5	F: GGAGAAGGTCTTCTCGGCCTC R: TTCAGAGAGCGAGACCCTGC	100
LMP1	F: TCTCCTTTGGCTCCTCCTGT R: TCGGTAGCTTGTTGAGGGTG	117
EBNA1	F: CCACAATGTCGTCTTACACC R: ATAACAGACAATGGACTCCCT	213

## Data Availability

Not applicable.

## Supplementary Materials

The following supporting information can be downloaded at: https://www.mdpi.com/article/10.3390/molecules27144666/s1, Table S1: The top 100 up-regulated and down-regulated proteins in cell lines treated with andrographolide and NaB. Table S2: The common up-regulated proteins in EBV-positive cells treated with andrographolide and NaB. Table S3: The functions of protein-related with histone modification processes. Figure S1: Andrographolide repressed EBV Zta expression. Figure S2: Western blot and densitometry reading/intensity ratio of Zta protein in cells treated with andrographolide.

## Sample Availability

Samples of the andrographolides are available from the authors.
