# Supplementary material for: Andrographolide Inhibits Epstein–Barr Virus Lytic Reactivation in EBV-Positive Cancer Cell Lines through the Modulation of Epigenetic-Related Proteins
Source: Molecules. 2022 Jul 21;27(14):4666. doi: 10.3390/molecules27144666 (PMC9316603; doi:10.3390/molecules27144666)
Supplement: Supplementary file 1 [file molecules-27-04666-s001.zip › molecules-1758662-supplementary.pdf]

Table S1. The top 100 up-regulated and down-regulated proteins in cell lines treated with andrographolide and NaB.

Entry	Entrez ID	Gene name	Mol. weight [kDa]	Sequence length	Q-value	Score	Intensity			Log2(FC)
							Untreated	NaB	Androg+NaB	Androg+NaB vs NaB
**P3HR1**										
Q9C0J1	79369	UDP-GlcNAc:betaGal beta-1,3-N-acetylglucosaminyltransferase 4(B3GNT4)	58.89	523	0.99	-2.80	0	0	3706600	21.82
P49848	6878	TATA-box binding protein associated factor 6(TAF6)	81.09	714	0.99	-34.72	0	0	2974400	21.50
O75683	6838	Surfeit 6(SURF6)	81.86	733	1.00	-3.40	0	0	2471200	21.24
P51815	7626	zinc finger protein 75D(ZNF75D)	83.64	729	0.99	-2.80	0	0	2471200	21.24
P23634	493	ATPase plasma membrane Ca2+ transporting 4(ATP2B4)	246.16	2206	0.98	-14.52	0	0	2445600	21.22
Q86TZ1	319089	tetratricopeptide repeat domain 6(TTC6)	74.51	675	0.99	-11.50	11258	0	2445600	21.22
O75791	9402	GRB2-related adaptor protein 2(GRAP2)	28.68	249	1.01	-4.24	0	0	2393000	21.19
Q9UKF7	26207	phosphatidylinositol transfer protein, cytoplasmic 1(PITPNC1)	64.19	571	1.00	-2.54	0	0	2172400	21.05
Q16696	1553	cytochrome P450 family 2 subfamily A member 13(CYP2A13)	9.12	80	0.99	-24.04	0	0	2007300	20.94
O14529	23316	cut like homeobox 2(CUX2)	573.83	5183	0.99	-25.84	37214	0	1988000	20.92
Q9BQE4	55829	selenoprotein S(SELENOS)	41.49	368	1.00	-7.24	0	0	1871200	20.84
Q96C01	84908	family with sequence similarity 136 member A(FAM136A)	79.93	747	1.01	-4.24	0	0	1739200	20.73
P16885	5336	phospholipase C gamma 2(PLCG2)	120.71	1064	0.99	-30.86	18690	0	1733000	20.72
Q96L12	125972	calreticulin 3(CALR3)	12.72	112	0.99	-31.14	0	0	1717400	20.71
P24928	5430	RNA polymerase II subunit A(POLR2A)	81.88	709	0.99	-2.01	0	0	1701600	20.70
Q7Z333	23064	senataxin(SETX)	58.51	504	0.98	-13.95	14633	0	1701600	20.70
Q8TCN5	22847	zinc finger protein 507(ZNF507)	100.67	876	0.99	-69.64	0	0	1640000	20.65
Q12948	2296	forkhead box C1(FOXC1)	122.33	1099	1.00	-7.38	0	0	1622600	20.63
Q9ULQ0	57464	striatin interacting protein 2(STRIP2)	506.27	4588	0.99	-48.04	0	0	1600000	20.61

Q16629	6432	serine and arginine rich splicing factor 7(SRSF7)	51.66	456	1.00	-2.54	0	0	1514900	20.53
Q96JF6	84622	zinc finger protein 594(ZNF594)	132.27	1211	0.99	-3.13	0	0	1474100	20.49
Q08AE8	56907	spire type actin nucleation factor 1(SPIRE1)	30.11	261	0.98	-12.80	0	0	1436500	20.45
Q147U1	162993	zinc finger protein 846(ZNF846)	128.76	1101	1.00	-3.73	0	0	1436500	20.45
Q8N6C5	3547	immunoglobulin superfamily member 1(IGSF1)	109.28	979	0.98	-12.61	119280	0	1316500	20.33
P53609	5229	protein geranylgeranyltransferase type I subunit beta(PGGT1B)	67.75	604	0.99	-20.17	0	0	1295000	20.30
Q07890	6655	SOS Ras/Rho guanine nucleotide exchange factor 2(SOS2)	161.57	1405	0.99	-24.26	627870	0	1252400	20.26
Q04759	5588	protein kinase C theta(PRKCQ)	87.13	740	1.01	-3.99	0	0	1252400	20.26
Q7Z406	79784	myosin heavy chain 14(MYH14)	65.17	563	1.01	-4.24	0	0	1215200	20.21
Q86TG7	23089	paternally expressed 10(PEG10)	133.28	1154	1.00	-6.23	25377	0	1145300	20.13
Q8IUQ4	6477	siah E3 ubiquitin protein ligase 1(SIAH1)	90.02	804	1.00	-2.54	0	0	1138600	20.12
Q9H7U1	54462	coiled-coil serine rich protein 2(CCSER2)	45.40	386	0.98	-12.80	0	0	1096400	20.06
P78363	24	ATP binding cassette subfamily A member 4(ABCA4)	91.15	800	0.99	-8.35	0	0	1076000	20.04
Q6ZUV0	344967	acyl-CoA thioesterase 7 pseudogene(LOC344967)	38.92	341	0.99	-3.43	0	0	1021200	19.96
O00566	10199	M-phase phosphoprotein 10(MPHOSPH10)	196.63	1668	0.99	-51.31	8334.3	0	1021200	19.96
P04234	915	CD3d molecule(CD3D)	49.10	442	1.00	-1.41	0	0	984530	19.91
Q8N1W1	64283	Rho guanine nucleotide exchange factor 28(ARHGEF28)	504.60	4544	0.99	-84.62	5863	0	967920	19.88
P28838	51056	leucine aminopeptidase 3(LAP3)	56.50	641	1.00	-1.41	0	0	916040	19.81
Q01970	5331	phospholipase C beta 3(PLCB3)	49.90	441	1.01	-4.91	12937	0	875980	19.74
O95602	25885	RNA polymerase I subunit A(POLR1A)	142.16	1253	1.00	-6.38	0	0	839890	19.68
Q96R72	283617	olfactory receptor family 4 subfamily K member 3 (gene/pseudogene)(OR4K3)	51.60	471	1.02	-0.60	0	0	836910	19.67
P03973	6590	secretory leukocyte peptidase inhibitor(SLPI)	53.99	495	1.01	-4.14	0	0	836910	19.67

Q3SY69	160428	aldehyde dehydrogenase 1 family member L2(ALDH1L2)	216.04	1871	1.00	-3.39	0	0	817760	19.64
P49641	4122	mannosidase alpha class 2A member 2(MAN2A2)	110.18	973	0.99	-10.33	0	0	747770	19.51
A3KN83	55206	strawberry notch homolog 1(SBNO1)	22.37	187	1.00	-5.70	0	0	717030	19.45
P0CB33	730291	zinc finger protein 735(ZNF735)	55.86	501	0.98	-11.19	0	0	700080	19.42
Q96PH1	79400	NADPH oxidase 5(NOX5)	195.46	1748	0.98	-13.55	0	0	671740	19.36
Q8N859	349075	zinc finger protein 713(ZNF713)	27.59	258	1.00	-6.23	330410	0	661950	19.34
P35789	81931	zinc finger protein 93(ZNF93)	53.54	484	1.00	-5.21	0	0	659540	19.33
Q5VW36	54914	focadhesin(FOCAD)	271.32	2390	0.99	-54.50	0	0	649460	19.31
Q9HA90	79825	EF-hand and coiled-coil domain containing 1(EFCC1)	45.73	432	0.98	-13.61	0	0	641770	19.29
O43929	5000	origin recognition complex subunit 4(ORC4)	37.91	330	1.01	-4.24	0	0	641770	19.29
Q9BSV6	79042	tRNA splicing endonuclease subunit 34(TSEN34)	142.51	1292	1.01	-4.24	0	0	641770	19.29
Q13099	8100	intraflagellar transport 88(IFT88)	46.74	409	1.00	-4.24	0	0	641770	19.29
Q5JWR5	23033	dopey family member 1(DOPEY1)	509.31	4462	0.99	-46.57	0	0	636430	19.28
Q86YD7	55138	family with sequence similarity 90 member A1(FAM90A1)	55.10	482	1.00	-6.95	55614	0	623690	19.25
P12111	1293	collagen type VI alpha 3 chain(COL6A3)	319.10	2863	0.99	-24.34	0	0	613800	19.23
Q9UGL9	54544	cysteine rich C-terminal 1(CRCT1)	52.74	450	0.99	-2.54	5162.1	0	610430	19.22
P14347	3783700	BFRF2(BFRF2)	39.87	351	1.01	-5.14	0	0	599800	19.19
Q8N8U2	124359	chromodomain Y-like 2(CDYL2)	7.30	68	0.98	-1.71	0	0	599800	19.19
Q66K80	284618	RUSC1 antisense RNA 1(RUSC1-AS1)	78.03	684	1.01	-4.95	9739.5	0	586210	19.16
Q5U4N7	554250	growth differentiation factor 5 opposite strand(GDF5OS)	72.06	697	0.98	-11.73	0	0	564110	19.11
Q03403	7032	trefoil factor 2(TFF2)	67.13	604	0.99	-10.26	211810	0	555710	19.08
Q86XG9	1.01E+08	neuroblastoma breakpoint family member 5, pseudogene(NBPF5P)	131.67	1242	1.00	-1.41	0	0	552830	19.08
Q9NPE2	51335	neugrin, neurite outgrowth associated(NGRN)	238.87	2109	0.99	-8.35	0	0	551300	19.07
P45880	7417	voltage dependent anion channel 2(VDAC2)	18.46	170	1.01	-4.24	0	0	551300	19.07

Q6ZMP0	79875	thrombospondin type 1 domain containing 4(THSD4)	114.22	1017	0.99	-33.20	0	0	536500	19.03
P33260	1562	cytochrome P450 family 2 subfamily C member 18(CYP2C18)	123.74	1097	0.99	-15.54	0	0	535120	19.03
Q4G0N8	285335	solute carrier family 9 member C1(SLC9C1)	58.44	531	0.98	-13.15	0	0	535120	19.03
Q9H6P5	55617	taspase 1(TASP1)	33.33	300	1.00	-3.80	0	0	535120	19.03
Q9UI10	8890	eukaryotic translation initiation factor 2B subunit delta(EIF2B4)	52.13	469	0.98	-11.29	0	0	482500	18.88
P40145	114	adenylate cyclase 8(ADCY8)	99.06	940	1.01	-4.15	0	0	479680	18.87
P13497	649	bone morphogenetic protein 1(BMP1)	138.02	1253	1.00	-4.24	0	0	479220	18.87
Q8NER5	130399	activin A receptor type 1C(ACVR1C)	96.67	861	1.00	-2.26	0	0	475680	18.86
Q9Y228	80342	TRAF3 interacting protein 3(TRAF3IP3)	117.06	1041	0.99	-9.77	0	0	475680	18.86
Q9UGC6	26575	regulator of G-protein signaling 17(RGS17)	378.06	3410	0.99	-17.40	0	0	451460	18.78
O15439	10257	ATP binding cassette subfamily C member 4(ABCC4)	54.52	491	0.98	-12.23	0	0	439200	18.74
Q9H0J9	64761	poly(ADP-ribose) polymerase family member 12(PARP12)	51.26	459	1.01	-3.74	0	0	423070	18.69
Q8N1G2	23070	cap methyltransferase 1(CMTR1)	103.91	926	0.99	-3.13	0	0	415840	18.67
Q8IZU1	171482	family with sequence similarity 9 member A(FAM9A)	112.48	1026	0.99	-11.49	0	0	415840	18.67
Q96SE7	84671	zinc finger protein 347(ZNF347)	532.22	4861	0.99	-37.52	0	0	415840	18.67
Q8NB15	118472	zinc finger protein 511(ZNF511)	85.65	747	1.00	-5.93	0	0	415840	18.67
A2RUC4	129450	tRNA-yW synthesizing protein 5(TYW5)	140.03	1234	1.00	-6.91	0	0	415840	18.67
F5HB39	4961479	tegument protein(ORF32)	57.01	493	1.01	-4.58	0	0	415090	18.66
Q15746	4638	myosin light chain kinase(MYLK)	175.66	1544	0.98	-15.62	0	0	404350	18.63
Q9NQP4	5203	prefoldin subunit 4(PFDN4)	28.81	245	0.97	-1.12	0	0	399700	18.61
A8MT19	646090	rhophilin Rho GTPase binding protein 2 pseudogene 1(RHPN2P1)	34.48	301	1.01	-4.24	0	0	393350	18.59
Q8NCM8	79659	dynein cytoplasmic 2 heavy chain 1(DYNC2H1)	378.02	3432	0.98	-9.15	5529.1	0	393350	18.59
Q9H1L0	128826	MIR1-1 host gene(MIR1-1HG)	616.62	5795	0.99	-17.70	0	0	391480	18.58
Q3KQV3	126375	zinc finger protein 792(ZNF792)	182.18	1603	0.99	-10.98	0	0	386160	18.56

Q2TAZ0	23130	autophagy related 2A(ATG2A)	65.54	581	0.99	-11.16	0	0	365520	18.48
O15265	6314	ataxin 7(ATXN7)	104.40	961	1.00	-5.44	0	0	365520	18.48
Q9NZ52	23163	golgi associated, gamma adaptin ear containing, ARF binding protein 3(GGA3)	34.21	305	1.00	-6.11	0	0	365520	18.48
Q6NZI2	284119	polymerase I and transcript release factor(PTRF)	215.40	1855	0.99	-17.40	0	0	362860	18.47
O60548	2306	forkhead box D2(FOXD2)	74.84	652	1.00	-6.12	0	0	360440	18.46
B7ZBB8	648791	protein phosphatase 1 regulatory subunit 3G(PPP1R3G)	135.17	1221	0.99	-2.37	0	0	347190	18.41
P51825	4299	AF4/FMR2 family member 1(AFF1)	277.35	2465	0.99	-46.59	7018.1	0	345250	18.40
Q68CQ1	374977	maestro heat like repeat family member 7(MROH7)	165.71	1445	1.00	-4.40	237550	0	345250	18.40
Q8WYQ9	23174	zinc finger CCHC-type containing 14(ZCCHC14)	133.55	1181	0.99	-20.93	0	0	345250	18.40
Q6P0N0	55320	MIS18 binding protein 1(MIS18BP1)	161.68	1486	0.98	-12.17	18767	0	345250	18.40
Q86VW2	115557	Rho guanine nucleotide exchange factor 25(ARHGEF25)	140.41	1243	1.00	-5.86	0	3697500	0	-21.82
Q2TBE0	143884	CWF19-like 2, cell cycle control (S. pombe)(CWF19L2)	22.13	199	1.01	-3.80	0	2673100	0	-21.35
Q9NZ20	50487	phospholipase A2 group III(PLA2G3)	86.72	756	0.98	-13.46	92974	2429400	0	-21.21
Q96NZ8	117166	WAP, follistatin/kazal, immunoglobulin, kunitz and netrin domain containing 1(WFIKKN1)	532.40	4646	0.99	-33.91	199980	2292500	0	-21.13
P49137	9261	mitogen-activated protein kinase-activated protein kinase 2(MAPKAPK2)	21.54	192	1.00	-4.24	0	2252600	0	-21.10
Q12893	11070	transmembrane protein 115(TMEM115)	12.32	100	1.00	-6.51	0	2243000	0	-21.10
P46777	6125	ribosomal protein L5(RPL5)	264.16	2414	1.00	-6.31	1004100	2176100	0	-21.05
Q6ZP82	285025	coiled-coil domain containing 141(CCDC141)	72.12	637	1.00	-7.39	0	2065800	0	-20.98
Q9UQ03	10391	coronin 2B(CORO2B)	61.66	555	1.00	-4.24	0	2065800	0	-20.98
P29317	1969	EPH receptor A2(EPHA2)	37.24	337	1.01	-3.80	0	2065800	0	-20.98
Q9H4G4	152007	GLI pathogenesis related 2(GLIPR2)	10.14	90	1.00	-4.24	0	2065800	0	-20.98
P0CG40	1E+08	Sp9 transcription factor(SP9)	44.46	420	1.00	-4.24	0	2065800	0	-20.98
Q6ZQV5	388507	zinc finger family member 788(ZNF788)	42.11	392	1.00	-4.24	0	2065800	0	-20.98

P32238	886	cholecystokinin A receptor(CCKAR)	113.98	1043	1.01	-4.99	0	2065800	0	-20.98
P51809	6845	vesicle associated membrane protein 7(VAMP7)	24.94	220	0.95	-0.75	0	1917700	0	-20.87
Q86V20	54537	family with sequence similarity 35 member A(FAM35A)	58.60	523	0.99	-10.30	0	1868100	0	-20.83
Q9NTZ6	10137	RNA binding motif protein 12(RBM12)	135.83	1284	1.00	-4.57	0	1484000	0	-20.50
Q07092	1307	collagen type XVI alpha 1 chain(COL16A1)	32.73	288	1.00	-3.80	0	1440700	0	-20.46
Q9NXA8	23408	sirtuin 5(SIRT5)	98.80	851	0.98	-9.12	17152	1405300	0	-20.42
Q9Y6N1	1353	COX11, cytochrome c oxidase copper chaperone(COX11)	17.26	153	0.98	-12.90	0	1405000	0	-20.42
Q9NYY3	10769	polo like kinase 2(PLK2)	56.68	494	1.00	-7.23	0	1405000	0	-20.42
Q8NDA2	256158	hemicentin 2(HMCN2)	305.48	2803	0.99	-36.74	69074	1282700	0	-20.29
Q9UGP8	11231	SEC63 homolog, protein translocation regulator(SEC63)	62.71	555	0.99	-28.55	0	1280800	0	-20.29
Q495X7	166655	tripartite motif containing 60(TRIM60)	118.17	1036	0.99	-9.43	125080	1233900	0	-20.23
Q93009	7874	ubiquitin specific peptidase 7(USP7)	52.63	481	1.01	-3.80	0	1171300	0	-20.16
Q8N4F0	80341	BPI fold containing family B member 2(BPIFB2)	71.79	673	0.99	-8.96	0	1167900	0	-20.16
Q05195	4084	MAX dimerization protein 1(MXD1)	81.10	757	1.00	-5.41	0	1052200	0	-20.00
A6NLX3	388333	speedy/RINGO cell cycle regulator family member E4(SPDYE4)	47.43	421	1.00	-1.20	0	1048300	0	-20.00
Q96M69	136332	leucine rich repeats and guanylate kinase domain containing(LRGUK)	62.69	534	0.99	-9.81	0	1048300	0	-20.00
O43752	10228	syntaxin 6(STX6)	446.70	4074	0.99	-16.72	0	1024400	0	-19.97
Q495N2	285641	solute carrier family 36 member 3(SLC36A3)	378.90	3392	0.99	-27.55	0	978120	0	-19.90
Q9UHB9	6730	signal recognition particle 68(SRP68)	147.34	1321	1.01	-3.80	289470	955520	0	-19.87
Q8IYS0	54762	GRAM domain containing 1C(GRAMD1C)	85.86	758	1.01	-3.80	7474800	935890	0	-19.84
Q9NQ03	85508	scratch family transcriptional repressor 2(SCRT2)	187.39	1684	0.98	-13.10	0	934370	0	-19.83
Q6NS38	121642	alkB homolog 2, alpha-ketoglutarate dependent dioxygenase(ALKBH2)	53.06	464	0.99	-3.13	0	934070	0	-19.83
Q8TF76	83903	germ cell associated 2, haspin(GSG2)	134.32	1204	1.00	-6.23	0	934070	0	-19.83

Q6GMV1	200810	ALG1, chitobiosyldiphosphodolichol beta-mannosyltransferase like(ALG1L)	41.95	388	0.99	-2.54	0	896920	0	-19.77
Q6ZW61	166379	Bardet-Biedl syndrome 12(BBS12)	68.56	610	1.00	-7.00	0	855760	0	-19.71
P47893	4995	olfactory receptor family 3 subfamily A member 2(OR3A2)	135.83	1284	1.00	-7.81	0	844040	0	-19.69
Q09472	2033	E1A binding protein p300(EP300)	61.19	540	0.99	-1.36	0	821710	0	-19.65
Q9NZI5	29841	grainyhead like transcription factor 1(GRHL1)	49.35	448	1.01	-3.76	0	793870	0	-19.60
Q3MIR4	161291	transmembrane protein 30B(TMEM30B)	15.16	138	0.98	-8.76	0	790480	0	-19.59
Q14781	84733	chromobox 2(CBX2)	71.16	623	1.00	-3.13	114530	754070	0	-19.52
Q6P179	64167	endoplasmic reticulum aminopeptidase 2(ERAP2)	41.02	365	1.00	-6.86	0	754070	0	-19.52
Q8NGP9	219493	olfactory receptor family 5 subfamily AR member 1 (gene/pseudogene)(OR5AR1)	58.07	501	1.01	-4.03	15928	754070	0	-19.52
P48449	4047	lanosterol synthase (2,3-oxidosqualene-lanosterol cyclase)(LSS)	36.22	320	1.00	-3.43	90075	713980	0	-19.45
Q9P016	29087	thymocyte nuclear protein 1(THYN1)	43.99	391	1.00	-3.80	13952000	713980	0	-19.45
Q9H310	57127	Rh family B glycoprotein (gene/pseudogene)(RHBG)	132.37	1210	1.00	-5.67	0	706120	0	-19.43
Q99567	4927	nucleoporin 88(NUP88)	107.85	985	1.01	-5.38	0	678380	0	-19.37
P06744	2821	glucose-6-phosphate isomerase(GPI)	33.00	293	1.01	-3.81	0	658700	0	-19.33
O75841	7348	uroplakin 1B(UPK1B)	83.82	783	1.00	-2.54	789980	652270	0	-19.32
P24723	5583	protein kinase C eta(PRKCH)	337.08	3075	0.99	-32.04	1418800	634900	0	-19.28
Q8TEF2	414152	chromosome 10 open reading frame 105(C10orf105)	169.84	1515	1.01	-3.92	0	623460	0	-19.25
Q9UPQ3	116987	ArfGAP with GTPase domain, ankyrin repeat and PH domain 1(AGAP1)	46.12	407	1.00	-7.15	0	623460	0	-19.25
Q9H0T7	64284	RAB17, member RAS oncogene family(RAB17)	133.62	1198	0.98	-13.65	0	622050	0	-19.25
Q9UPN4	22994	centrosomal protein 131(CEP131)	126.78	1202	1.00	-2.74	38086	608150	0	-19.21
A0A0B4J1Y8	28773	immunoglobulin lambda variable 9-49(IGLV9-49)	241.01	2156	0.99	-26.99	10484	602510	0	-19.20
Q5XUX0	79791	F-box protein 31(FBXO31)	54.17	520	0.99	-29.67	0	594730	0	-19.18

P05014	3441	interferon alpha 4(IFNA4)	33.25	296	0.99	-1.39	0	594730	0	-19.18
Q96JI7	80208	spastic paraplegia 11 (autosomal recessive)(SPG11)	22.77	202	0.99	-1.31	0	553310	0	-19.08
P28328	5828	peroxisomal biogenesis factor 2(PEX2)	134.28	1210	1.00	-2.54	0	530910	0	-19.02
Q14674	9700	extra spindle pole bodies like 1, separase(ESPL1)	241.61	2109	0.99	-26.37	29495	528310	0	-19.01
P07996	7057	thrombospondin 1(THBS1)	25.39	223	0.98	-1.71	0	509680	0	-18.96
Q6ZQQ6	83889	WD repeat domain 87(WDR87)	69.79	601	0.99	-29.40	0	503370	0	-18.94
P62263	6208	ribosomal protein S14(RPS14)	287.59	2564	0.99	-27.72	0	487410	0	-18.89
Q8TDY2	9821	RB1 inducible coiled-coil 1(RB1CC1)	304.79	2768	0.99	-42.62	730470	483820	0	-18.88
P0DMR3	6315	ATXN8 opposite strand (non-protein coding)(ATXN8OS)	82.02	713	0.99	-24.46	3093700	480980	0	-18.88
Q96Q89	9585	kinesin family member 20B(KIF20B)	86.61	775	0.99	-21.42	0	453610	0	-18.79
Q9NTJ3	10051	structural maintenance of chromosomes 4(SMC4)	73.10	665	0.99	-2.01	0	441030	0	-18.75
Q3ZN06			276.17	2471	0.99	-25.08	0	432370	0	-18.72
P16083	4835	NAD(P)H quinone dehydrogenase 2(NQO2)	164.21	1484	0.98	-1.71	0	429890	0	-18.71
Q96QZ0	116337	pannexin 3(PANX3)	98.53	844	0.98	-11.84	0	429890	0	-18.71
Q5JTH9	23223	ribosomal RNA processing 12 homolog(RRP12)	137.46	1288	1.00	-1.41	0	427880	0	-18.71
Q9HCJ0	57690	trinucleotide repeat containing 6C(TNRC6C)	208.53	1909	0.99	-26.13	6081.5	427880	0	-18.71
Q96PZ0	54517	pseudouridylate synthase 7 (putative)(PUS7)	56.22	512	0.99	-2.01	0	425620	0	-18.70
Q92569	8503	phosphoinositide-3-kinase regulatory subunit 3(PIK3R3)	122.91	1123	0.98	-12.38	0	419270	0	-18.68
P08913	150	adrenoceptor alpha 2A(ADRA2A)	99.15	858	0.99	-17.38	4639.5	414760	0	-18.66
Q8TBZ5	91392	zinc finger protein 502(ZNF502)	531.78	4684	0.99	-71.37	70677	410520	0	-18.65
Q8IZU0	171483	family with sequence similarity 9 member B(FAM9B)	87.60	763	0.99	-9.48	0	402680	0	-18.62
Q9Y315	51071	deoxyribose-phosphate aldolase(DERA)	60.97	534	1.01	-3.88	0	390340	0	-18.57
Q9Y644	5986	RFNG O-fucosylpeptide 3-beta-N-acetylglucosaminyltransferase(RFNG)	234.71	2061	0.99	-24.54	0	372000	0	-18.50

A6NFT4	387885	cilia and flagella associated protein 73(CFAP73)	135.26	1187	1.00	-7.60	0	371520	0	-18.50
Q9UBP4	27122	dickkopf WNT signaling pathway inhibitor 3(DKK3)	226.94	2016	0.99	-31.02	0	370630	0	-18.50
Q16385	6757	SSX family member 2(SSX2)	89.63	810	0.99	-2.91	595020	351930	0	-18.42
Q96QT6	57649	PHD finger protein 12(PHF12)	84.44	766	0.99	-21.09	0	348070	0	-18.41
P02768	213	albumin(ALB)	35.70	317	0.98	-8.77	0	342820	0	-18.39
O60674	3717	Janus kinase 2(JAK2)	360.27	3174	0.98	-13.67	9042	342310	0	-18.38
Q495C1	285498	ring finger protein 212(RNF212)	138.11	1213	0.99	-26.71	100110	332630	0	-18.34
Q9Y252	6049	ring finger protein 6(RNF6)	104.94	926	0.99	-18.72	8346.7	332630	0	-18.34
P09234	6631	small nuclear ribonucleoprotein polypeptide C(SNRPC)	14.33	122	0.99	-10.28	0	332630	0	-18.34
O75056	9672	syndecan 3(SDC3)	117.31	1086	0.99	-33.44	52393	319240	0	-18.28
A6NHJ4	344787	zinc finger protein 860(ZNF860)	99.71	900	1.00	-3.39	0	316430	0	-18.27
Q96AY2	146956	essential meiotic structure-specific endonuclease 1(EME1)	12.25	105	0.99	-1.20	0	315450	0	-18.27
O75054	3321	immunoglobulin superfamily member 3(IGSF3)	75.23	658	0.99	-9.44	0	310120	0	-18.24
P48029	6535	solute carrier family 6 member 8(SLC6A8)	39.02	350	0.99	-3.13	0	308080	0	-18.23
Q8IUB3	280664	WAP four-disulfide core domain 10B(WFDC10B)	18.62	163	1.00	-8.04	48384	306190	0	-18.22
B0I1T2	64005	myosin IG(MYO1G)	73.13	665	0.99	-1.20	0	302010	0	-18.20
Q9BWC9	29903	coiled-coil domain containing 106(CCDC106)	109.88	963	0.99	-11.39	0	300490	0	-18.20
P51449	6097	RAR related orphan receptor C(RORC)	59.61	520	0.99	-29.00	58192	297140	0	-18.18
Q9ULW8	51702	peptidyl arginine deiminase 3(PADI3)	90.97	807	0.99	-10.29	600230	291030	0	-18.15
Q6P5S2	352999	chromosome 6 open reading frame 58(C6orf58)	70.15	609	1.00	-6.56	0	289400	0	-18.14

AGS-EBV

Q8N2S1	8425	latent transforming growth factor beta binding protein 4(LTBP4)	107.91	988	0.99	-50.28	0	0	3443500	21.72
Q9H6D7	54930	HAUS augmin like complex subunit 4(HAUS4)	41.16	373	0.99	-2.01	0	0	2275300	21.12

Q9UBN7	10013	histone deacetylase 6(HDAC6)	37.40	330	0.99	-8.88	0	0	2275300	21.12
Q96N06	124045	spermatogenesis associated 33(SPATA33)	16.43	146	1.00	-1.39	0	0	2203500	21.07
Q96IR2	91664	zinc finger protein 845(ZNF845)	103.97	911	0.99	-20.65	0	0	1871000	20.84
P56557	757	transmembrane protein 50B(TMEM50B)	104.22	903	0.99	-9.69	11213	0	1850800	20.82
P18615	7936	negative elongation factor complex member E(NELFE)	20.49	175	1.00	-2.54	0	0	1839000	20.81
Q92685	10195	ALG3, alpha-1,3-mannosyltransferase(ALG3)	90.15	838	0.98	-1.71	0	0	1813600	20.79
O75829	11061	leukocyte cell derived chemotaxin 1(LECT1)	241.58	2109	1.09	-0.60	0	0	1804500	20.78
O43290	9092	squamous cell carcinoma antigen recognized by T-cells 1(SART1)	56.04	494	1.00	-5.86	0	0	1804500	20.78
Q8WTZ3	7652	zinc finger protein 99(ZNF99)	35.55	315	1.00	-5.95	32909	0	1804500	20.78
Q8IVL1	89797	neuron navigator 2(NAV2)	275.48	2570	0.99	-18.81	72466	0	1747200	20.74
Q16654	5166	pyruvate dehydrogenase kinase 4(PDK4)	191.48	1782	1.00	-6.93	0	0	1655800	20.66
P53420	1286	collagen type IV alpha 4 chain(COL4A4)	135.62	1261	1.00	-6.53	0	0	1488100	20.51
Q92738	9712	USP6 N-terminal like(USP6NL)	53.25	465	0.98	-11.92	0	0	1460100	20.48
Q9Y3S1	65268	WNK lysine deficient protein kinase 2(WNK2)	247.00	2156	0.99	-16.22	0	0	1445700	20.46
I3L1E1	147646	chromosome 19 open reading frame 84(C19orf84)	29.62	264	1.00	-3.80	0	0	1431300	20.45
Q16254	1874	E2F transcription factor 4(E2F4)	37.53	341	1.01	-3.80	0	0	1431300	20.45
A8MPX8	151649	protein phosphatase 2C like domain containing 1(PP2D1)	59.53	520	1.00	-4.40	0	0	1431300	20.45
Q8WYR4	89765	radial spoke head 1 homolog(RSPH1)	150.59	1343	0.99	-8.99	0	0	1431300	20.45
O14492	10603	SH2B adaptor protein 2(SH2B2)	57.22	492	0.98	-12.70	0	0	1431300	20.45
Q8WWF8	133690	calcyphosine like(CAPSL)	173.10	1573	0.99	-9.20	0	0	1397900	20.41
P57071	63977	PR/SET domain 15(PRDM15)	88.00	745	0.98	-13.41	0	0	1397900	20.41
Q8N141	284406	ZFP82 zinc finger protein(ZFP82)	21.78	200	1.00	-2.54	0	0	1397900	20.41
Q9P2N7	90293	kelch like family member 13(KLHL13)	51.61	447	1.00	-8.35	0	0	1344700	20.36

P09884	5422	DNA polymerase alpha 1, catalytic subunit(POLA1)	16.81	142	1.00	-5.21	33311	0	1344700	20.36
Q12870	6939	transcription factor 15 (basic helix-loop-helix)(TCF15)	27.34	258	0.99	-8.86	0	0	1262400	20.27
Q14315	2318	filamin C(FLNC)	613.38	5635	0.99	-20.78	0	0	1245700	20.25
Q7M4L6	90525	Src homology 2 domain containing F(SHF)	68.02	593	1.00	-3.66	0	0	1141300	20.12
Q9BZI7	65109	UPF3 regulator of nonsense transcripts homolog B (yeast)(UPF3B)	280.01	2419	0.99	-21.31	0	0	1079500	20.04
Q8NGC5	79549	olfactory receptor family 6 subfamily J member 1 (gene/pseudogene)(OR6J1)	98.91	870	1.00	-8.12	0	0	1067200	20.03
Q8NBK3	285362	sulfatase modifying factor 1(SUMF1)	245.87	2179	0.98	-12.23	0	0	1067200	20.03
Q03164	4297	lysine methyltransferase 2A(KMT2A)	335.86	3164	0.99	-8.90	0	0	1036500	19.98
P49189	223	aldehyde dehydrogenase 9 family member A1(ALDH9A1)	62.64	543	0.99	-2.54	0	0	1022400	19.96
Q13117	57055	deleted in azoospermia 2(DAZ2)	54.99	486	0.99	-2.54	0	0	1022400	19.96
Q9Y2I6	22981	ninein like(NINL)	94.47	828	0.99	-11.01	0	0	1022400	19.96
A0A0U1RR11	389857	centromere protein V like 1(CENPVL1)	93.49	819	1.01	-4.92	0	0	1017100	19.96
O60313	4976	OPA1, mitochondrial dynamin like GTPase(OPA1)	132.37	1188	1.01	-3.80	0	0	1017100	19.96
Q9UI47	29119	catenin alpha 3(CTNNA3)	19.28	175	1.00	-1.41	0	0	953620	19.86
Q9BPX1	51171	hydroxysteroid 17-beta dehydrogenase 14(HSD17B14)	46.91	423	1.00	-1.43	0	0	953620	19.86
Q86UC2	83861	radial spoke 3 homolog(RSPH3)	22.76	211	1.09	-0.60	7390	0	901920	19.78
Q07001	1144	cholinergic receptor nicotinic delta subunit(CHRND)	75.71	694	1.00	-3.14	0	0	901890	19.78
O14545	10906	TRAF-type zinc finger domain containing 1(TRAFD1)	30.11	266	0.99	-2.80	0	0	846370	19.69
Q9ULC8	29801	zinc finger DHHC-type containing 8(ZDHHC8)	91.93	832	1.01	-3.85	0	0	797120	19.60
P35611	118	adducin 1(ADD1)	37.33	334	1.00	-4.28	0	0	776230	19.57
P13747	3133	major histocompatibility complex, class I, E(HLA-E)	72.83	647	0.99	-36.31	0	0	757280	19.53
Q6UXH1	79174	cysteine rich with EGF like domains 2(CRELD2)	112.99	1043	0.99	-26.14	0	0	737320	19.49

P36382	2702	gap junction protein alpha 5(GJA5)	90.04	805	1.00	-4.24	0	0	737320	19.49
O43148	8731	RNA guanine-7 methyltransferase(RNMT)	20.62	182	1.01	-4.24	0	0	737320	19.49
Q92621	23165	nucleoporin 205(NUP205)	131.69	1186	1.00	-5.23	0	0	730660	19.48
Q08708	10871	CD300c molecule(CD300C)	316.41	2701	0.99	-24.27	0	0	726380	19.47
Q6PJP8	9937	DNA cross-link repair 1A(DCLRE1A)	59.91	528	1.01	-5.14	39343	0	691060	19.40
Q2M5E4	431704	regulator of G-protein signaling 21(RGS21)	17.67	152	0.99	-2.26	0	0	676910	19.37
O43593	55806	hair growth associated(HR)	206.94	1896	1.00	-6.72	0	0	661880	19.34
Q5T1M5	23307	FK506 binding protein 15(FKBP15)	112.26	1020	0.98	-1.71	0	0	659010	19.33
Q6XYQ8	341359	synaptotagmin 10(SYT10)	71.00	643	0.99	-46.62	0	0	638490	19.28
Q9ULE3	27147	DENN domain containing 2A(DENND2A)	79.14	683	0.99	-23.97	0	0	632150	19.27
Q6P995	165215	family with sequence similarity 171 member B(FAM171B)	96.62	853	0.97	-0.85	0	0	625160	19.25
Q02952	9590	A-kinase anchoring protein 12(AKAP12)	169.27	1507	0.98	-15.83	0	0	618540	19.24
Q9BVQ7	79029	spermatogenesis associated 5 like 1(SPATA5L1)	36.73	328	1.00	-6.78	0	0	600530	19.20
Q9H190	27111	syndecan binding protein 2(SDCBP2)	122.76	1070	0.96	-1.12	0	0	591450	19.17
Q9NYU1	55757	UDP-glucose glycoprotein glucosyltransferase 2(UGGT2)	56.06	478	1.00	-5.37	0	0	586780	19.16
Q01484	287	ankyrin 2(ANK2)	85.06	765	0.99	-10.57	0	0	572850	19.13
P19387	5432	RNA polymerase II subunit C(POLR2C)	282.58	2492	0.99	-64.44	0	0	559370	19.09
Q96M63	93233	coiled-coil domain containing 114(CCDC114)	63.53	561	1.00	-4.24	0	0	551650	19.07
Q5JXM2	728464	methyltransferase like 24(METTL24)	147.16	1264	0.99	-14.29	12717	0	551650	19.07
Q8TE04	53354	pantothenate kinase 1(PANK1)	122.11	1114	0.99	-9.36	0	0	551650	19.07
P28864	1487917	type 1 membrane protein; possible membrane fusogen; binds cell surface heparan sulphate; involved in cell entry; involved in cell-to-cell spread(U39)	547.84	5150	0.99	-58.33	36213	0	550890	19.07

Q8TE85	57822	grainyhead like transcription factor 3(GRHL3)	47.09	418	1.03	-0.60	0	0	537160	19.03
Q6TDP4	339451	kelch like family member 17(KLHL17)	72.22	645	0.99	-24.98	21687	0	516960	18.98
Q2TAY7	55234	DNA replication regulator and spliceosomal factor(SMU1)	245.27	2191	0.99	-2.01	0	0	503060	18.94
Q8IXQ4	55425	GPALPP motifs containing 1(GPALPP1)	14.52	151	1.00	-1.45	0	0	494410	18.92
Q80943	1403640	replication protein E1(E1)	80.32	711	0.98	-14.53	0	0	485160	18.89
Q9HB07	60314	chromosome 12 open reading frame 10(C12orf10)	35.02	299	1.01	-4.91	0	0	479150	18.87
P19438	7132	TNF receptor superfamily member 1A(TNFRSF1A)	178.70	1556	1.00	-8.04	0	0	468470	18.84
Q9NS39	105	adenosine deaminase, RNA specific B2 (inactive)(ADARB2)	111.69	1017	1.00	-7.38	30635	0	466650	18.83
Q9NSQ0	91695	ribosomal RNA processing 7 homolog B, pseudogene(RRP7BP)	67.26	620	1.00	-4.24	0	0	466650	18.83
Q96RP7	79690	galactose-3-O-sulfotransferase 4(GAL3ST4)	194.86	1747	1.01	-5.14	0	0	462190	18.82
Q8NB91	2187	Fanconi anemia complementation group B(FANCB)	171.23	1495	0.99	-20.25	0	0	438100	18.74
Q96BT3	80152	centromere protein T(CENPT)	158.05	1392	0.99	-14.16	0	0	435780	18.73
Q9UKM7	11253	mannosidase alpha class 1B member 1(MAN1B1)	87.45	743	0.99	-2.25	11463	0	430350	18.72
Q4ZHG4	84624	fibronectin type III domain containing 1(FNDC1)	33.74	294	0.99	-2.80	0	0	420210	18.68
Q9Y2W7	30818	potassium voltage-gated channel interacting protein 3(KCNIP3)	183.30	1647	1.00	-3.39	42621	0	411900	18.65
P41229	8242	lysine demethylase 5C(KDM5C)	61.96	556	0.99	-10.29	0	0	397180	18.60
P17010	7543	zinc finger protein, X-linked(ZFX)	136.06	1226	0.99	-9.38	60652	0	396380	18.60
Q9Y2L1	22894	DIS3 homolog, exosome endoribonuclease and 3'-5' exoribonuclease(DIS3)	208.88	1883	0.99	-15.94	38958	0	386390	18.56
Q68CQ1	374977	maestro heat like repeat family member 7(MROH7)	165.71	1445	1.00	-4.40	0	0	385050	18.55
Q8NEK5	147694	zinc finger protein 548(ZNF548)	50.02	435	1.01	-4.10	0	0	373670	18.51
P89471			117.01	1074	0.98	-11.92	0	0	372570	18.51

Q6ZMZ3	161176	spectrin repeat containing nuclear envelope family member 3(SYNE3)	99.81	895	1.00	-6.41	0	0	368770	18.49
Q8TC71	132671	spermatogenesis associated 18(SPATA18)	38.16	356	1.01	-4.24	0	0	363820	18.47
Q5T2D3	23252	OTU deubiquitinase 3(OTUD3)	50.53	463	1.00	-8.03	0	0	361710	18.46
Q9UFE4	339829	coiled-coil domain containing 39(CCDC39)	191.98	1665	0.99	-20.83	0	0	356110	18.44
Q13131	5562	protein kinase AMP-activated catalytic subunit alpha 1(PRKAA1)	51.57	464	1.00	-1.20	0	0	348930	18.41
Q9BYH8	64332	NFKB inhibitor zeta(NFKBIZ)	32.83	289	1.00	-5.97	0	0	340480	18.38
Q9NV06	25879	DDB1 and CUL4 associated factor 13(DCAF13)	43.38	376	0.95	-0.85	0	0	332930	18.34
A6NHM9	1E+08	monooxygenase, DBH-like 2, pseudogene(MOXD2P)	78.65	712	0.98	-1.05	0	0	332930	18.34
P0C7W0	92340	proline rich 29(PRR29)	58.28	527	0.99	-1.41	0	0	329740	18.33
Q5K651	54809	sterile alpha motif domain containing 9(SAMD9)	46.02	426	1.00	-7.74	0	0	328830	18.33
Q9H3R2	56667	mucin 13, cell surface associated(MUC13)	23.07	208	0.99	-2.01	0	0	327850	18.32
Q8NHV4	121441	neural precursor cell expressed, developmentally down-regulated 1(NEDD1)	10.96	94	0.99	-2.80	0	4437300	0	-22.08
Q8IWC1	79649	MAP7 domain containing 3(MAP7D3)	36.03	316	1.00	-7.60	830090	4085400	0	-21.96
Q8N1W2	374655	zinc finger protein 710(ZNF710)	43.81	393	1.01	-3.80	0	3968600	0	-21.92
Q96IP4	55603	family with sequence similarity 46 member A(FAM46A)	101.28	919	1.00	-3.40	244310	3489600	0	-21.73
Q13443	8754	ADAM metallopeptidase domain 9(ADAM9)	475.98	4158	0.99	-43.27	49025	3320100	0	-21.66
P60468	10952	Sec61 translocon beta subunit(SEC61B)	231.60	2033	0.99	-24.10	0	2974000	0	-21.50
Q92771	440081	DEAD/H-box helicase 12, pseudogene(DDX12P)	68.89	592	0.98	-11.66	0	2946800	0	-21.49
Q9UKX3	8735	myosin heavy chain 13(MYH13)	48.44	429	0.99	-19.01	2511200	2535100	0	-21.27
Q9NQG5	58490	regulation of nuclear pre-mRNA domain containing 1B(RPRD1B)	61.29	563	0.99	-2.01	1926500	2535100	0	-21.27

A6H8Y1	55814	B double prime 1, subunit of RNA polymerase III transcription initiation factor IIIB(BDP1)	335.92	2997	0.99	-44.18	0	2330700	0	-21.15
P61550	1.05E+08	endogenous retrovirus group S71 member 1 Env polyprotein(LOC105372315)	113.79	1012	1.00	-6.72	0	2209200	0	-21.08
Q15051	9657	IQ motif containing B1(IQCB1)	256.72	2319	0.98	-14.01	0	2190500	0	-21.06
Q09013	1760	dystrophia myotonica protein kinase(DMPK)	145.65	1323	1.00	-5.27	81065	2086200	0	-20.99
O75581	4040	LDL receptor related protein 6(LRP6)	94.63	844	1.00	-6.62	45172	2081200	0	-20.99
Q8NCE0	80746	tRNA splicing endonuclease subunit 2(TSEN2)	53.25	465	0.94	-0.60	71752	1901300	0	-20.86
Q8NGC1	390439	olfactory receptor family 11 subfamily G member 2(OR11G2)	77.50	676	1.01	-3.80	13315	1893000	0	-20.85
Q96SZ4	84891	zinc finger and SCAN domain containing 10(ZSCAN10)	34.31	304	1.00	-5.65	0	1881000	0	-20.84
Q13190	6811	syntaxin 5(STX5)	25.18	227	1.00	-2.26	0	1646600	0	-20.65
Q6GMV3	391356	peptidyl-tRNA hydrolase domain containing 1(PTRHD1)	57.60	503	1.00	-1.20	1226000	1399600	0	-20.42
Q9Y4E5	26036	zinc finger protein 451(ZNF451)	58.05	534	0.99	-1.73	0	1378300	0	-20.39
O75800	51364	zinc finger MYND-type containing 10(ZMYND10)	15.65	142	1.00	-5.51	0	1271600	0	-20.28
Q9NR16	283316	CD163 molecule like 1(CD163L1)	175.49	1599	0.99	-25.78	95874	1165300	0	-20.15
Q9Y291	51650	mitochondrial ribosomal protein S33(MRPS33)	107.53	999	1.00	-4.28	0	1102700	0	-20.07
O95104	57466	SR-related CTD associated factor 4(SCAF4)	65.42	552	1.00	-2.54	0	1102700	0	-20.07
O60347	23232	TBC1 domain family member 12(TBC1D12)	62.57	542	1.00	-6.76	0	1102700	0	-20.07
Q9H857	64943	5'-nucleotidase domain containing 2(NT5DC2)	63.52	568	1.00	-7.92	0	1049900	0	-20.00
Q9H7M6	65249	zinc finger SWIM-type containing 4(ZSWIM4)	72.89	651	1.00	-6.55	0	1049900	0	-20.00
P78406	8480	ribonucleic acid export 1(RAE1)	64.41	557	1.01	-3.88	118540	1036400	0	-19.98
Q0VF49	1.01E+08	KIAA2012(KIAA2012)	593.38	5537	0.99	-31.37	0	1006300	0	-19.94

Q96CD0	55336	F-box and leucine rich repeat protein 8(FBXL8)	119.23	1052	0.99	-11.39	0	948210	0	-19.85
O95897	93145	olfactomedin 2(OLFM2)	515.49	4599	0.99	-202.53	123660	909330	0	-19.79
O60268	9764	KIAA0513(KIAA0513)	132.95	1204	1.00	-2.26	0	891850	0	-19.77
Q1L5Z9	164832	LON peptidase N-terminal domain and ring finger 2(LONRF2)	96.86	858	1.01	-4.02	0	891850	0	-19.77
P23025	7507	XPA, DNA damage recognition and repair factor(XPA)	87.95	802	0.99	-2.26	0	891850	0	-19.77
Q9UNP9	10450	peptidylprolyl isomerase E(PPIE)	76.00	685	1.00	-3.13	0	886660	0	-19.76
Q70SY1	64764	cAMP responsive element binding protein 3 like 2(CREB3L2)	87.20	808	1.00	-6.56	0	876830	0	-19.74
Q01524	1671	defensin alpha 6(DEFA6)	81.66	739	0.99	-10.03	40317	854440	0	-19.70
Q8WUX2	494143	ChaC cation transport regulator homolog 2(CHAC2)	101.55	887	1.00	-5.97	0	842510	0	-19.68
P10635	1565	cytochrome P450 family 2 subfamily D member 6(CYP2D6)	81.44	765	0.99	-18.59	0	809040	0	-19.63
Q9HBL8	57407	NmrA like redox sensor 1(NMRAL1)	52.73	454	1.00	-3.13	0	780340	0	-19.57
Q5JSZ5	84726	proline rich coiled-coil 2B(PRRC2B)	531.78	4684	0.99	-46.68	119590	748690	0	-19.51
Q9Y4I1	4644	myosin VA(MYO5A)	131.85	1198	0.98	-13.57	14329	739390	0	-19.50
Q9HCH0	57701	NCK associated protein 5 like(NCKAP5L)	28.61	250	1.00	-7.85	0	739390	0	-19.50
O43295	9901	SLIT-ROBO Rho GTPase activating protein 3(SRGAP3)	43.65	407	1.00	-1.41	0	739390	0	-19.50
P35942			95.11	846	0.99	-9.85	48389	739390	0	-19.50
Q9P1A6	9228	DLG associated protein 2(DLGAP2)	119.11	1093	1.00	-2.80	0	724740	0	-19.47
O14926	25794	fascin actin-bundling protein 2, retinal(FSCN2)	87.61	775	0.99	-2.37	0	705320	0	-19.43
P47710	1446	casein alpha s1(CSN1S1)	250.68	2198	0.99	-18.84	0	699890	0	-19.42
Q496A3	221409	spermatogenesis associated serine rich 1(SPATS1)	46.87	416	0.97	-1.12	0	681750	0	-19.38
Q5VIR6	55275	VPS53, GARP complex subunit(VPS53)	26.93	240	1.00	-1.12	0	681750	0	-19.38
Q53EZ4	55165	centrosomal protein 55(CEP55)	130.16	1153	0.99	-19.31	0	667380	0	-19.35
Q86VP3	23241	phosphofurin acidic cluster sorting protein 2(PACS2)	138.34	1230	0.99	-38.26	0	661550	0	-19.34

P30414	4820	natural killer cell triggering receptor(NKTR)	133.77	1257	0.99	-16.51	35511	656380	0	-19.32
O60216	5885	RAD21 cohesin complex component(RAD21)	112.60	1052	0.99	-2.54	0	642410	0	-19.29
P36578	6124	ribosomal protein L4(RPL4)	85.74	793	0.99	-9.74	0	636520	0	-19.28
Q5PT55	347051	solute carrier family 10 member 5(SLC10A5)	229.87	2025	0.99	-44.81	99438	624080	0	-19.25
O95810	8436	serum deprivation response(SDPR)	18.76	172	1.01	-5.40	0	607020	0	-19.21
Q99469	6769	SH3 and cysteine rich domain(STAC)	85.89	752	1.00	-5.51	2280000	580050	0	-19.15
O95935	9096	T-box 18(TBX18)	180.97	1627	1.00	-2.61	0	567640	0	-19.11
Q08397	4016	lysyl oxidase like 1(LOXL1)	76.26	666	1.00	-4.28	0	557300	0	-19.09
Q96PP9	115361	guanylate binding protein 4(GBP4)	72.70	649	1.00	-4.24	0	550870	0	-19.07
O15067	5198	phosphoribosylformylglycinamidine synthase(PFAS)	91.35	816	0.99	-41.52	319110	549900	0	-19.07
P36507	5605	mitogen-activated protein kinase kinase 2(MAP2K2)	59.14	525	1.00	-7.34	0	549150	0	-19.07
Q9Y672	29929	ALG6, alpha-1,3-glucosyltransferase(ALG6)	79.42	708	1.01	-4.85	0	543940	0	-19.05
Q9NZN4	30846	EH domain containing 2(EHD2)	193.41	1762	0.99	-22.66	0	543940	0	-19.05
Q8NFF5	80308	flavin adenine dinucleotide synthetase 1(FLAD1)	81.44	765	1.01	-3.80	0	543940	0	-19.05
Q8WZA1	55624	protein O-linked mannose N-acetylglucosaminyltransferase 1 (beta 1,2-)(POMGNT1)	42.37	380	1.00	-4.24	0	543940	0	-19.05
Q13033	29966	striatin 3(STRN3)	82.86	757	0.99	-28.82	0	543940	0	-19.05
Q9NVP1	8886	DEAD-box helicase 18(DDX18)	41.99	362	1.00	-2.26	0	539020	0	-19.04
Q9BZV3	50939	interphotoreceptor matrix proteoglycan 2(IMPG2)	218.97	1942	1.00	-3.37	0	539020	0	-19.04
Q96LT9	55599	RNA binding region (RNP1, RRM) containing 3(RNPC3)	305.41	2715	0.99	-10.72	96821	532190	0	-19.02
O60911	1515	cathepsin V(CTSV)	149.56	1327	0.98	-14.91	0	531860	0	-19.02
Q13620	8450	cullin 4B(CUL4B)	81.96	732	1.00	-4.24	0	510780	0	-18.96
Q9NZM3	50618	intersectin 2(ITSN2)	462.52	4303	0.99	-100.34	0	510780	0	-18.96
B2RBV5	93622	Morf4 family associated protein 1 like 1 pseudogene(LOC93622)	39.05	334	1.00	-3.80	1797600	510780	0	-18.96

P33908	4121	mannosidase alpha class 1A member 1(MAN1A1)	78.66	672	0.98	-9.58	0	510780	0	-18.96
Q13087	64714	protein disulfide isomerase family A member 2(PDIA2)	49.44	430	1.00	-5.89	992360	510780	0	-18.96
Q9YLQ9			22.17	210	1.01	-4.24	0	510780	0	-18.96
Q5TB80	22832	centrosomal protein 162(CEP162)	105.06	963	1.01	-4.24	0	484330	0	-18.89
Q6ZP80	130827	transmembrane protein 182(TMEM182)	60.39	546	0.99	-14.06	990030	484330	0	-18.89
Q9GZX9	57045	twisted gastrulation BMP signaling modulator 1(TWSG1)	4.68	41	1.00	-4.24	0	484330	0	-18.89
Q9Y446	11187	plakophilin 3(PKP3)	128.01	1094	0.98	-12.88	163350	473360	0	-18.85
Q9NQX6	55422	zinc finger protein 331(ZNF331)	81.54	740	1.00	-6.34	0	458800	0	-18.81
P68443			94.12	809	0.98	-15.15	0	457710	0	-18.80
P52529			101.26	916	1.01	-0.60	14946	453010	0	-18.79
P35998	5701	proteasome 26S subunit, ATPase 2(PSMC2)	271.32	2390	0.99	-30.12	30352	449260	0	-18.78
Q9C0A1	85446	zinc finger homeobox 2(ZFHX2)	71.90	646	1.00	-3.43	0	447060	0	-18.77
Q02763	7010	TEK receptor tyrosine kinase(TEK)	38.09	349	0.99	-51.68	0	438020	0	-18.74
B5MCY1	1E+08	tudor domain containing 15(TDRD15)	378.48	3412	0.99	-33.72	0	427110	0	-18.70
O75426	26261	F-box protein 24(FBXO24)	60.59	528	1.00	-4.26	0	415140	0	-18.66
O95995	2622	growth arrest specific 8(GAS8)	53.03	463	1.00	-3.13	0	409260	0	-18.64
P21439	5244	ATP binding cassette subfamily B member 4(ABCB4)	17.47	154	0.98	-14.17	0	403710	0	-18.62
Q9Y4B6	9730	DDB1 and CUL4 associated factor 1(DCAF1)	133.85	1235	0.98	-15.02	0	403710	0	-18.62
P59901	23547	leukocyte immunoglobulin like receptor A4(LILRA4)	54.95	480	1.00	-4.24	0	403710	0	-18.62
Q96S97	91663	myeloid associated differentiation marker(MYADM)	112.45	1018	0.99	-33.19	0	403710	0	-18.62
Q9Y5H7	56143	protocadherin alpha 5(PCDHA5)	206.89	1849	0.99	-28.41	0	403710	0	-18.62
Q96L92	81609	sorting nexin family member 27(SNX27)	103.17	946	0.98	-11.79	816900	403710	0	-18.62
P59044	171389	NLR family pyrin domain containing 6(NLRP6)	113.28	1006	0.99	-3.13	0	401800	0	-18.62
Q96BY7	55102	autophagy related 2B(ATG2B)	40.17	350	1.00	-1.43	0	399540	0	-18.61

Q9NZH6	27178	interleukin 37(IL37)	42.97	386	1.00	-4.34	812020	399110	0	-18.61

HON1-EBV

Q5TAA0	55001	tetratricopeptide repeat domain 22(TTC22)	85.79	779	0.99	-2.80	0	0	3317900	21.66
Q8N300	374969	small vasohibin binding protein(SVBP)	158.21	1423	1.00	-7.23	0	0	2358400	21.17
Q8N7W2	222389	BEN domain containing 7(BEND7)	20.71	189	0.99	-3.13	0	0	1783600	20.77
Q8N6N2	148014	tetratricopeptide repeat domain 9B(TTC9B)	64.41	557	0.98	-15.65	0	0	1565300	20.58
O15119	6926	T-box 3(TBX3)	225.03	2004	0.99	-23.20	0	0	1475600	20.49
P51814	7592	zinc finger protein 41(ZNF41)	102.05	936	0.99	-2.01	0	0	1475600	20.49
Q96T37	64783	RNA binding motif protein 15(RBM15)	65.70	598	0.98	-14.62	0	0	1436600	20.45
Q9GZZ7	64096	GDNF family receptor alpha 4(GFRA4)	278.86	2443	0.99	-11.43	0	0	1272600	20.28
Q86UQ4	154664	ATP binding cassette subfamily A member 13(ABCA13)	67.66	600	1.00	-2.26	0	0	1272400	20.28
P30566	158	adenylosuccinate lyase(ADSL)	73.01	626	0.99	-10.63	0	0	1272400	20.28
Q13443	8754	ADAM metallopeptidase domain 9(ADAM9)	475.98	4158	0.99	-43.27	0	0	1043400	19.99
Q330K2	137682	NADH:ubiquinone oxidoreductase complex assembly factor 6(NDUFAF6)	77.67	698	0.99	-2.01	0	0	1016200	19.95
Q96A19	92922	coiled-coil domain containing 102A(CCDC102A)	141.69	1278	0.99	-2.87	0	0	967200	19.88
Q9NZI7	7342	upstream binding protein 1 (LBP-1a)(UBP1)	69.86	611	0.99	-2.01	0	0	967200	19.88
P43080	2978	guanylate cyclase activator 1A(GUCA1A)	27.00	233	1.00	-1.61	0	0	934200	19.83
Q6ZQQ6	83889	WD repeat domain 87(WDR87)	69.79	601	0.99	-29.40	17949	0	934200	19.83
Q15735	27124	inositol polyphosphate-5-phosphatase J(INPP5J)	46.94	402	0.98	-15.14	0	0	902000	19.78
B2RBV5	93622	Morf4 family associated protein 1 like 1 pseudogene(LOC93622)	39.05	334	1.00	-3.80	0	0	829620	19.66
O95427	23556	phosphatidylinositol glycan anchor biosynthesis class N(PIGN)	139.99	1265	0.99	-16.88	195140	0	802840	19.61
Q9P031	29080	coiled-coil domain containing 59(CCDC59)	98.93	860	1.01	-4.03	0	0	760490	19.54

Q06945	6659	SRY-box 4(SOX4)	54.87	493	0.99	-24.88	0	0	729900	19.48
Q9P281	57597	BAH domain and coiled-coil containing 1(BAHCC1)	114.65	1066	0.99	-28.14	0	0	713530	19.44
Q8WW38	23414	zinc finger protein, FOG family member 2(ZFPM2)	35.97	310	0.99	-9.36	0	0	710210	19.44
O60911	1515	cathepsin V(CTSV)	149.56	1327	0.98	-14.91	175100	0	660550	19.33
P08700	3562	interleukin 3(IL3)	530.25	4857	0.99	-20.35	13610	0	648090	19.31
Q9H330	23731	transmembrane protein 245(TMEM245)	29.38	254	1.01	-4.92	0	0	640350	19.29
Q9UL62	7224	transient receptor potential cation channel subfamily C member 5(TRPC5)	124.20	1124	0.99	-14.07	0	0	640350	19.29
Q9H9Y2	80135	ribosome production factor 1 homolog(RPF1)	94.68	850	0.99	-16.40	0	0	630120	19.27
Q6U7Q0	79692	zinc finger protein 322(ZNF322)	78.58	662	0.98	-8.77	0	0	630120	19.27
Q9UM54	4646	myosin VI(MYO6)	52.97	465	0.99	-10.26	0	0	611360	19.22
Q96Q11	51095	tRNA nucleotidyl transferase 1(TRNT1)	104.15	922	1.00	-4.24	0	0	605390	19.21
Q92478	9976	C-type lectin domain family 2 member B(CLEC2B)	70.59	627	0.98	-13.54	0	0	595000	19.18
Q03519	6891	transporter 2, ATP binding cassette subfamily B member(TAP2)	47.85	436	1.00	-4.24	0	0	581550	19.15
Q7Z572	374955	spermatogenesis associated 21(SPATA21)	54.44	519	0.99	-1.48	0	0	579400	19.14
Q9H8E8	57325	lysine acetyltransferase 14(KAT14)	48.28	419	0.99	-11.41	0	0	570510	19.12
P10323	49	acrosin(ACR)	242.67	2297	0.99	-10.16	0	0	566740	19.11
Q96S38	26750	ribosomal protein S6 kinase C1(RPS6KC1)	25.71	227	0.99	-2.80	0	0	555650	19.08
Q8TAB7	137196	CCDC26 long non-coding RNA(CCDC26)	23.35	211	0.99	-3.13	0	0	545060	19.06
Q12772	6721	sterol regulatory element binding transcription factor 2(SREBF2)	51.80	459	0.99	-3.13	3905.4	0	460740	18.81
Q9NZV7	23619	zinc finger imprinted 2(ZIM2)	330.46	3051	0.99	-29.73	0	0	418030	18.67
P57077	56911	MAP3K7 C-terminal like(MAP3K7CL)	41.86	375	0.99	-8.64	293300	0	406250	18.63
Q5TB80	22832	centrosomal protein 162(CEP162)	105.06	963	1.01	-4.24	0	0	405230	18.63
Q96PD2	131566	discoidin, CUB and LCCL domain containing 2(DCBLD2)	229.83	2036	1.00	-3.53	0	0	403810	18.62
Q9UC06	7621	zinc finger protein 70(ZNF70)	63.47	579	1.00	-6.26	0	0	396560	18.60

Q6NUN9	155061	zinc finger protein 746(ZNF746)	180.43	1613	1.00	-6.60	0	0	395830	18.59
Q9HCI5	57692	MAGE family member E1(MAGEE1)	28.57	259	1.00	-4.24	0	0	385970	18.56
P35942			95.11	846	0.99	-9.85	11540	0	356610	18.44
Q8N187	79800	calcium responsive transcription factor(CARF)	58.74	523	1.00	-3.63	0	0	342610	18.39
Q86WI3	84166	NLR family CARD domain containing 5(NLRC5)	56.06	490	0.99	-2.54	0	0	336250	18.36
Q9H5K3	84197	protein-O-mannose kinase(POMK)	6.04	51	1.01	-4.24	0	0	329530	18.33
P20309	1131	cholinergic receptor muscarinic 3(CHRM3)	75.96	661	0.99	-9.38	0	0	328690	18.33
P52355			67.78	610	0.98	-15.80	0	0	328690	18.33
Q9NRC9	56914	otoraplin(OTOR)	59.86	542	1.00	-2.12	0	0	308950	18.24
Q5H9L2	340543	transcription elongation factor A like 5(TCEAL5)	80.49	715	0.99	-22.25	0	0	308750	18.24
Q86Y56	54919	dynein axonemal assembly factor 5(DNAAF5)	163.03	1435	1.00	-2.54	0	0	308260	18.23
Q7L5Y6	55070	de-etiolated homolog 1 (Arabidopsis)(DET1)	316.05	2804	0.99	-41.18	0	0	303270	18.21
P51504	7634	zinc finger protein 80(ZNF80)	177.97	1602	1.00	-5.67	0	0	287470	18.13
P11245	10	N-acetyltransferase 2(NAT2)	63.22	577	0.98	-12.56	0	0	285700	18.12
Q92698	8438	RAD54-like (S. cerevisiae)(RAD54L)	68.93	598	1.00	-1.41	0	0	283020	18.11
Q96JK4	84439	HHIP like 1(HHIPL1)	87.39	830	0.99	-46.51	0	0	280820	18.10
O60437	5493	periplakin(PPL)	75.75	663	0.99	-16.17	0	0	276700	18.08
Q15628	8717	TNFRSF1A associated via death domain(TRADD)	56.36	478	1.00	-5.75	0	0	273770	18.06
Q13200	5708	proteasome 26S subunit, non-ATPase 2(PSMD2)	24.01	207	1.00	-6.34	0	0	271100	18.05
Q03468	2074	ERCC excision repair 6, chromatin remodeling factor(ERCC6)	88.62	754	0.99	-17.18	0	0	271040	18.05
Q86T82	57695	ubiquitin specific peptidase 37(USP37)	304.81	2723	0.99	-25.01	225680	0	270610	18.05
Q9UBG7	11317	recombination signal binding protein for immunoglobulin kappa J region like(RBPJL)	15.16	135	1.00	-3.43	0	0	264640	18.01
P17544	11016	activating transcription factor 7(ATF7)	79.78	709	0.99	-2.80	0	0	257180	17.97

P13584	1580	cytochrome P450 family 4 subfamily B member 1(CYP4B1)	158.54	1429	1.00	-8.00	6187.1	0	256680	17.97
P48454	5533	protein phosphatase 3 catalytic subunit gamma(PPP3CC)	192.09	1690	0.99	-10.29	0	0	254380	17.96
P51991	220988	heterogeneous nuclear ribonucleoprotein A3(HNRNPA3)	303.10	2845	0.99	-30.42	0	0	252980	17.95
Q9UN86	9908	G3BP stress granule assembly factor 2(G3BP2)	38.62	369	1.00	-4.24	0	0	251750	17.94
Q9H7V2	79953	synapse differentiation inducing 1(SYNDIG1)	73.58	660	0.99	-23.20	8977.8	0	249700	17.93
A8MX76	440854	calpain 14(CAPN14)	140.92	1279	0.99	-77.02	0	0	248450	17.92
Q8IXT2	63946	DMRT like family C2(DMRTC2)	47.56	418	0.98	-12.86	1183800	0	234320	17.84
Q92900	5976	UPF1, RNA helicase and ATPase(UPF1)	74.08	674	0.99	-1.55	0	0	232740	17.83
O75038	9651	phospholipase C eta 2(PLCH2)	213.86	1854	0.99	-11.34	85149	0	228380	17.80
O94991	26050	SLIT and NTRK like family member 5(SLITRK5)	28.92	249	0.99	-11.47	0	0	227150	17.79
Q14738	5528	protein phosphatase 2 regulatory subunit B'delta(PPP2R5D)	286.10	2527	1.00	-3.43	0	0	221490	17.76
Q5JTH9	23223	ribosomal RNA processing 12 homolog(RRP12)	127.00	1132	0.98	-14.80	0	0	217570	17.73
Q711Q0	118461	chromosome 10 open reading frame 71(C10orf71)	50.23	439	0.99	-3.13	0	0	212260	17.70
Q8IVM7	283487	long intergenic non-protein coding RNA 346(LINC00346)	15.57	137	0.99	-2.80	0	0	211340	17.69
O75309	1014	cadherin 16(CDH16)	30.79	267	1.00	-6.98	0	0	208450	17.67
Q02641	782	calcium voltage-gated channel auxiliary subunit beta 1(CACNB1)	299.61	2752	0.99	-20.12	0	0	205280	17.65
Q9ULD9	57507	zinc finger protein 608(ZNF608)	222.52	1989	0.98	-14.18	0	0	204710	17.64
P23025	7507	XPA, DNA damage recognition and repair factor(XPA)	87.95	802	0.99	-2.26	0	0	202670	17.63
Q14135	9686	vestigial like family member 4(VGLL4)	80.39	725	1.00	-5.66	0	0	200010	17.61
Q86VY4	85453	TSPY like 5(TSPYL5)	25.38	218	1.00	-3.62	0	0	191830	17.55
Q6RW13	57085	angiotensin II receptor associated protein(AGTRAP)	76.47	682	1.00	-3.66	0	0	190710	17.54

Q8WX92	25920	negative elongation factor complex member B(NELFB)	199.75	1778	1.00	-3.63	0	0	190710	17.54
A6NNM8	440307	tubulin tyrosine ligase like 13, pseudogene(TTLL13P)	237.93	2057	0.99	-10.58	75143	0	190710	17.54
Q99832	10574	chaperonin containing TCP1 subunit 7(CCT7)	86.11	757	0.99	-9.06	0	0	189920	17.54
P15088	1359	carboxypeptidase A3(CPA3)	35.82	318	1.01	-4.24	0	0	186790	17.51
Q5SRE7	254295	phytanoyl-CoA dioxygenase domain containing 1(PHYHD1)	68.93	622	0.99	-20.46	18813	0	177620	17.44
Q9BXP5	51593	serrate, RNA effector molecule(SRRT)	40.66	358	1.00	-6.19	0	0	177620	17.44
Q7Z713	353322	ankyrin repeat domain 37(ANKRD37)	71.43	664	0.98	-12.32	0	0	177170	17.43
O60725	23463	isoprenylcysteine carboxyl methyltransferase(ICMT)	38.42	362	0.99	-29.73	0	0	176910	17.43
P29122	5046	proprotein convertase subtilisin/kexin type 6(PCSK6)	201.56	1792	0.99	-37.34	6478.6	0	174310	17.41
Q14573	3710	inositol 1,4,5-trisphosphate receptor type 3(ITPR3)	28.28	248	0.99	-8.63	0	0	173190	17.40
P50213	3419	isocitrate dehydrogenase 3 (NAD(+)) alpha(IDH3A)	130.84	1200	0.98	-10.89	0	0	172450	17.40
Q5TA82	353141	late cornified envelope 2D(LCE2D)	80.47	702	1.00	-4.44	0	0	172450	17.40
Q8NEF3	153733	coiled-coil domain containing 112(CCDC112)	54.30	481	1.00	-2.54	0	7002400	0	-22.74
Q8NG48	55180	lines homolog 1(LINS1)	135.20	1177	0.98	-8.78	0	5145200	0	-22.29
Q9UGP4	8994	LIM domains containing 1(LIMD1)	65.70	580	0.98	-13.54	0	4878500	0	-22.22
Q8NFT8	92737	delta/notch like EGF repeat containing(DNER)	87.79	805	1.00	-6.23	0	1657300	0	-20.66
Q8NGQ1	283189	olfactory receptor family 9 subfamily G member 4(OR9G4)	15.88	137	0.99	-3.13	0	1657300	0	-20.66
Q4G0N8	285335	solute carrier family 9 member C1(SLC9C1)	58.44	531	0.98	-13.15	0	1269500	0	-20.28
Q6JQN1	80724	acyl-CoA dehydrogenase family member 10(ACAD10)	53.50	476	1.00	-1.41	0	1198100	0	-20.19
Q8TDQ0	84868	hepatitis A virus cellular receptor 2(HAVCR2)	83.57	761	1.00	-3.73	0	985550	0	-19.91
Q9BXY0	84549	MAK16 homolog(MAK16)	154.54	1374	0.99	-19.51	0	985550	0	-19.91

O15544	23434	long intergenic non-protein coding RNA 1565(LINC01565)	84.34	761	0.99	-2.87	0	977230	0	-19.90
Q8IWD4	150275	coiled-coil domain containing 117(CCDC117)	32.36	275	1.00	-5.97	0	955870	0	-19.87
P51813	660	BMX non-receptor tyrosine kinase(BMX)	57.63	508	0.99	-2.01	0	955490	0	-19.87
Q9BW62	84056	katanin catalytic subunit A1 like 1(KATNAL1)	21.52	205	1.00	-3.43	0	861530	0	-19.72
Q9ULL8	57477	shroom family member 4(SHROOM4)	104.82	951	1.00	-7.43	6798.8	858120	0	-19.71
P0DMR3	6315	ATXN8 opposite strand (non-protein coding)(ATXN8OS)	40.76	370	1.00	-2.54	0	797670	0	-19.61
P08047	6667	Sp1 transcription factor(SP1)	79.19	711	1.01	-3.80	86754	775578	0	-19.56
Q9BTV5	79187	fibronectin type III and SPRY domain containing 1(FSD1)	48.95	428	0.99	-9.99	0	763910	0	-19.54
Q86VQ3	84203	thioredoxin domain containing 2(TXNDC2)	83.98	738	0.99	-39.48	0	742530	0	-19.50
O75916	8787	regulator of G-protein signaling 9(RGS9)	116.18	1012	1.00	-5.16	0	723720	0	-19.47
Q9NWT1	55003	PAK1 interacting protein 1(PAK1IP1)	20.47	178	0.99	-10.62	0	675890	0	-19.37
B0I1T2	64005	myosin IG(MYO1G)	73.13	665	0.99	-1.20	108930	660300	0	-19.33
Q6PGP7	9652	tetratricopeptide repeat domain 37(TTC37)	119.60	1066	0.99	-32.27	20173	642190	0	-19.29
Q8NHJ6	11006	leukocyte immunoglobulin like receptor B4(LILRB4)	504.60	4544	0.99	-170.90	463010	640950	0	-19.29
Q9Y4E5	26036	zinc finger protein 451(ZNF451)	58.05	534	0.99	-1.73	0	621900	0	-19.25
P02462	1282	collagen type IV alpha 1 chain(COL4A1)	164.88	1464	0.99	-35.91	0	618620	0	-19.24
P04062	2629	glucosylceramidase beta(GBA)	26.11	221	1.01	-3.80	0	607020	0	-19.21
Q9P015	29088	mitochondrial ribosomal protein L15(MRPL15)	17.76	157	1.00	-3.52	0	597930	0	-19.19
Q96J66	85320	ATP binding cassette subfamily C member 11(ABCC11)	51.92	443	0.98	-11.27	32533	568750	0	-19.12
A6H8Y1	55814	B double prime 1, subunit of RNA polymerase III transcription initiation factor IIIB(BDP1)	335.92	2997	0.99	-44.18	4907.8	568750	0	-19.12
P36807	1496946	early protein(E6)	99.06	917	0.99	-2.87	0	568750	0	-19.12

Q13045	2314	FLII, actin remodeling protein(FLII)	77.67	703	0.98	-12.78	0	568750	0	-19.12
P17030	219749	zinc finger protein 25(ZNF25)	77.52	671	0.99	-19.24	0	546520	0	-19.06
P61371	3670	ISL LIM homeobox 1(ISL1)	47.35	421	0.99	-2.26	0	542100	0	-19.05
Q9Y672	29929	ALG6, alpha-1,3-glucosyltransferase(ALG6)	79.42	708	1.01	-4.85	5373.1	530330	0	-19.02
Q8N8D1	10081	programmed cell death 7(PDCD7)	33.40	299	1.01	-4.24	0	530330	0	-19.02
O14994	8224	synapsin III(SYN3)	74.54	665	0.99	-9.62	240350	465540	0	-18.83
Q9BW92	80222	threonyl-tRNA synthetase 2, mitochondrial (putative)(TARS2)	52.68	450	0.99	-20.01	143890	465010	0	-18.83
Q52MB2	387856	coiled-coil domain containing 184(CCDC184)	38.59	344	1.00	-4.24	0	462160	0	-18.82
Q8NFC6	259282	biorientation of chromosomes in cell division 1 like 1(BOD1L1)	94.38	835	0.99	-2.01	0	459470	0	-18.81
Q5QGZ9	160364	C-type lectin domain family 12 member A(CLEC12A)	221.41	1911	0.99	-2.54	0	448900	0	-18.78
Q6UWY2	400668	protease, serine 57(PRSS57)	143.23	1233	0.99	-24.20	31934	448900	0	-18.78
Q5T481	282996	RNA binding motif protein 20(RBM20)	142.59	1288	0.99	-40.48	198940	440370	0	-18.75
Q9NPC6	51778	myozenin 2(MYOZ2)	75.35	642	1.00	-6.23	0	432840	0	-18.72
Q9Y6H8	2700	gap junction protein alpha 3(GJA3)	161.10	1454	0.99	-2.54	0	411370	0	-18.65
O95379	25816	TNF alpha induced protein 8(TNFAIP8)	221.67	2063	0.99	-14.31	0	409150	0	-18.64
A8MW92	51105	PHD finger protein 20-like 1(PHF20L1)	31.37	279	1.00	-4.24	0	401420	0	-18.61
A0A0C4DH29	28473	immunoglobulin heavy variable 1-3(IGHV1-3)	32.11	288	1.00	-6.56	0	401170	0	-18.61
Q13183	9058	solute carrier family 13 member 2(SLC13A2)	68.74	595	0.99	-8.44	139750	401170	0	-18.61
P38435	2677	gamma-glutamyl carboxylase(GGCX)	165.19	1462	0.99	-9.87	0	397480	0	-18.60
P0CG32	644353	zinc finger CCHC-type containing 18(ZCCHC18)	327.82	2946	0.98	-14.23	0	393680	0	-18.59
Q99715	1303	collagen type XII alpha 1 chain(COL12A1)	51.84	450	0.99	-23.80	0	393010	0	-18.58
O94955	22836	Rho related BTB domain containing 3(RHOBTB3)	35.09	315	1.00	-2.80	0	381170	0	-18.54
P59901	23547	leukocyte immunoglobulin like receptor A4(LILRA4)	54.95	480	1.00	-4.24	0	368550	0	-18.49
Q99795	10223	glycoprotein A33(GPA33)	96.70	843	0.99	-2.54	0	351610	0	-18.42

O75791	9402	GRB2-related adaptor protein 2(GRAP2)	28.68	249	1.01	-4.24	1311500	349730	0	-18.42
P35968	3791	kinase insert domain receptor(KDR)	166.57	1382	0.99	-30.87	90205	344230	0	-18.39
Q96N77	121274	zinc finger protein 641(ZNF641)	68.26	625	1.00	-6.86	57523	339660	0	-18.37
Q9H3R5	64946	centromere protein H(CENPH)	45.75	392	1.00	-8.25	0	319890	0	-18.29
K9N5Q8			226.37	1988	0.99	-9.02	0	319060	0	-18.28
P32019	3633	inositol polyphosphate-5-phosphatase B(INPP5B)	572.01	5405	0.99	-20.72	0	318450	0	-18.28
Q9UNX4	10885	WD repeat domain 3(WDR3)	160.16	1388	0.99	-19.25	0	313410	0	-18.26
Q5W064	142910	lipase family member J(LIPJ)	253.92	1943	0.99	-24.25	0	309060	0	-18.24
Q9UIK4	23604	death associated protein kinase 2(DAPK2)	132.49	1205	0.99	-8.67	0	307770	0	-18.23
O95751	23641	leucine zipper down-regulated in cancer 1(LDOC1)	43.09	371	0.98	-14.84	0	307770	0	-18.23
Q2YD98	57654	UV stimulated scaffold protein A(UVSSA)	35.63	319	0.99	-2.54	0	298260	0	-18.19
Q9HCZ1	55713	zinc finger protein 334(ZNF334)	76.87	697	0.99	-2.01	0	296820	0	-18.18
Q8N3T6	92293	transmembrane protein 132C(TMEM132C)	61.25	536	0.98	-10.84	76775	290420	0	-18.15
Q96M95	146849	coiled-coil domain containing 42(CCDC42)	23.64	206	1.01	-3.80	0	289340	0	-18.14
P52848	3340	N-deacetylase and N-sulfotransferase 1(NDST1)	93.02	824	0.99	-21.95	4014.8	283180	0	-18.11
Q9UBL9	22953	purinergic receptor P2X 2(P2RX2)	40.93	354	0.99	-2.80	0	277380	0	-18.08
Q9H6X2	84168	anthrax toxin receptor 1(ANTXR1)	51.67	471	1.00	-2.26	0	274840	0	-18.07
O60268	9764	KIAA0513(KIAA0513)	110.70	1021	1.00	-2.54	0	274840	0	-18.07
C9JE40	197135	PAT1 homolog 2(PATL2)	276.54	2458	0.99	-34.92	39694	273530	0	-18.06
O75683	6838	surfeit 6(SURF6)	81.86	733	1.00	-3.40	1607000	271600	0	-18.05
O75129	23245	astrotactin 2(ASTN2)	50.82	463	0.99	-8.58	104180	269290	0	-18.04
P08473	4311	membrane metalloendopeptidase(MME)	168.89	1498	0.99	-23.88	430100	265730	0	-18.02
Q96IP4	55603	family with sequence similarity 46 member A(FAM46A)	101.28	919	1.00	-3.40	10991	264870	0	-18.01
Q96KN9	219770	gap junction protein delta 4(GJD4)	51.32	457	1.00	-1.20	0	263060	0	-18.01

P61011	6729	signal recognition particle 54(SRP54)	160.25	1430	0.98	-14.34	166990	259720	0	-17.99
Q6TDU7	55259	cancer susceptibility candidate 1(CASC1)	278.16	2602	0.98	-10.87	0	256490	0	-17.97
Q9UPM6	26468	LIM homeobox 6(LHX6)	255.94	2273	0.99	-1.41	0	255830	0	-17.96
Q5VST6	51104	abhydrolase domain containing 17B(ABHD17B)	25.02	223	1.00	-3.63	0	252560	0	-17.95
Q15063	10631	periostin(POSTN)	61.04	540	0.99	-2.01	0	248640	0	-17.92
O14713	9270	integrin subunit beta 1 binding protein 1(ITGB1BP1)	115.69	1053	1.00	-3.14	170340	247910	0	-17.92
Q8NHV1	168537	GTPase, IMAP family member 7(GIMAP7)	173.35	1551	0.98	-15.67	0	246800	0	-17.91
Q8TD90	139599	MAGE family member E2(MAGEE2)	49.13	471	1.00	-1.41	0	242450	0	-17.89
Q8N7B1	150280	HORMA domain containing 2(HORMAD2)	112.22	975	1.00	-6.41	84317	240910	0	-17.88
P55786	9520	aminopeptidase puromycin sensitive(NPEPPS)	162.02	1434	0.99	-16.98	71810	238100	0	-17.86
Q16540	6150	mitochondrial ribosomal protein L23(MRPL23)	16.94	152	1.09	-0.60	100770	236570	0	-17.85
O95180	8912	calcium voltage-gated channel subunit alpha1 H(CACNA1H)	65.10	583	0.99	-2.54	0	234830	0	-17.84
Q96K49	84910	transmembrane protein 87B(TMEM87B)	149.85	1356	0.99	-27.08	9761.9	233580	0	-17.83
Q9Y210	7225	transient receptor potential cation channel subfamily C member 6(TRPC6)	42.79	387	1.01	-4.85	0	233580	0	-17.83
P19224	54578	UDP glucuronosyltransferase family 1 member A6(UGT1A6)	35.74	317	1.00	-4.24	0	233580	0	-17.83
Q9Y3S2	27309	zinc finger protein 330(ZNF330)	195.91	1684	0.98	-8.77	0	233580	0	-17.83
Q96LQ0	145376	protein phosphatase 1 regulatory subunit 36(PPP1R36)	44.31	381	1.00	-3.80	0	233350	0	-17.83
Q04538	940442	polyprotein(POWVgp1)	70.90	626	0.99	-10.31	0	233260	0	-17.83
Q9H4E5	57381	ras homolog family member J(RHOJ)	83.63	746	1.00	-6.94	76725	233260	0	-17.83
Q8WVC0	123169	LEO1 homolog, Paf1/RNA polymerase II complex component(LEO1)	149.56	1327	0.99	-38.21	49865	231870	0	-17.82
Q9Y4C4	9258	malignant fibrous histiocytoma amplified sequence 1(MFHAS1)	30.37	266	1.00	-6.79	0	230480	0	-17.81

Q9P227	57636	Rho GTPase activating protein 23(ARHGAP23)	15.53	137	0.99	-24.56	0	230450	0	-17.81

Table S2. The common up-regulated proteins in EBV-positive cells treated with andrographolide and NaB.

Entrez ID	Uniport ID	Protein	symbol	P3FR1					AGS-EBV					HONE1-EBV				
				Intensity			Log2(Intensity)		Intensity			Log2(Intensity)		Intensity			Log2(Intensity)	
				Untreated	NaB	NAB + Androg	NaB	NAB + Androg	Untreated	NaB	NAB + Androg	NaB	NAB +Androg	Untreated	NaB	NAB +Androg	NaB	NAB +Androg
493	P23634	ATPase plasma membrane Ca^{2+} transporting 4	ATP2B4	0	0	2445600	0.00	21.22175703	0	0	22595	0.00	14.46	0.00	0.00	56803	0	15.79
1557	P33261	Cytochrome P450 family 2 subfamily C member 19	CYP2C19	0	0	6188.8	0.00	12.60	0	0	3575.4	0.00	11.80	0.00	0.00	11670	0	13.51
1832	P15924	Desmoplakin	DSP	0	0	106580	0.00	16.70	0	0	7895.6	0.00	12.95	0.00	0.00	80015	0	16.29
2162	P00488	Coagulation factor XIII A chain	F13A1	7159.4	0	153950	-12.81	4.43	0	0	18820	0.00	14.20	0.00	0.00	60297	0	15.88
4001	P20700	Lamin B1	LMNB1	0	0	37167	0.00	15.18	0	0	5536.1	0.00	12.43	0.00	0.00	36034	0	15.14
4299	P51825	AF4/FMR2 family member 1	AFF1	7018.1	0	345250	-12.78	5.62	0	0	14791	0.00	13.85	0.00	0.00	2911.9	0	11.51
4670	P52272	Heterogeneous nuclear ribonucleoprotein M	HNRNPM	0	0	52958	0	15.69256102	0	0	17360	0.00	14.08	0.00	0.00	49947	0	15.61
5073	O95453	Poly(A)-specific ribonuclease	PARN	0	0	7341.8	0.00	12.84	0	0	18981	0.00	14.21	0.00	0.00	38791	0	15.24
7621	Q9UC06	Zinc finger protein 70	ZNF70	0	0	20740	0.00	14.34	0	0	31988	0.00	14.97	0.00	0.00	396560	0	18.60
9344	Q9UL54	TAO kinase 2	TAOK2	0	0	13996	0.00	13.77	0	0	17095	0.00	14.06	0.00	0.00	98337	0	16.59

9815	Q14161	GIT ArfGAP 2	GIT2	10234	0	135886.2	-13.32	3.73	0	0	14671	0.00	13.84	0.00	0.00	21616	0	14.40
10294	O60884	DnaJ heat shock protein family (Hsp40) member A2	DNAJA2	0	0	32608	0.00	14.99	0	0	86677	0.00	16.40	0.00	0.00	4100.2	0	12.00
22981	Q9Y2I6	Ninein like	NINL	0	0	13802	0.00	13.75	0	0	1022400	0.00	19.96	0.00	0.00	24076	0	14.56
26095	Q4KMQ1	Protein tyrosine Phosphatase, non-receptor type 20	PTPN20	0	0	137360	0.00	17.07	0	0	3121.3	0.00	11.61	0.00	0.00	5732.3	0	12.48
54476	Q9NWF9	Ring finger protein 216	RNF216	0	0	315410	0.00	18.27	0	0	25835	0.00	14.66	0.00	0.00	36507	0	15.16
55012	Q969Q6	Protein phosphatase 2 regulatory subunit B''gamma	PPP2R3C	6566.2	0	148926.7	-12.68	4.50	68245	0	397135	-16.06	2.54	0.00	0.00	23312	0	14.51
55081	Q9NWB7	Intraflagellar transport 57	IFT57	0	0	2904.7	0.00	11.50	0	0	10571	0.00	13.37	0.00	0.00	16873	0.00	14.04
56950	Q9NRG4	SET and MYND domain containing 2	SMYD2	0	0	117640	0.00	16.84	51299	0	343579.8	-15.65	2.74	0.00	0.00	28231	0	14.78
57716	Q9BXM0	Periaxin	PRX	0	0	6268.5	0.00	12.61	0	0	114000	0.00	16.80	0.00	0.00	4207.4	0	12.04
64283	Q8N1W1	Rho guanine nucleotide exchange factor 28	ARHGEF28	5863	0	967920	-12.52	19.88	0	0	63988	0.00	15.97	0.00	0.00	10382	0	13.34
65078	Q9BZR6	Reticulon 4 receptor	RTN4R	15960	0	760064	-13.96	5.57	0	0	243120	0.00	17.89	0.00	0.00	14225	0	13.80
79778	Q8IY33	MICAL like 2	MICALL2	0	0	10454	0.00	13.35	0	0	52632	0.00	15.68	0.00	0.00	7326.9	0	12.84
80824	Q9BY84	Dual specificity phosphatase 16	DUSP16	26138	0	396920	-14.67	3.92	0	0	14818	0.00	13.86	3354.50	0.00	14670	-11.711882	4.37

83786	Q9BZ67	FERM domain containing 8	FRMD8	0	0	38807	0.00	15.24	0	0	2596.5	0.00	11.34	0.00	0.00	16510	0	14.01
84439	Q96JK4	HHIP like 1	HHIPL1	0	0	184790	0.00	17.50	0	0	39490	0.00	15.27	0.00	0.00	280820	0	18.10
92400	Q96H35	RNA binding motif protein 18	RBM18	0	0	9993.7	0.00	13.29	0	0	15723	0.00	13.94	10288.00	0.00	24210	-13.328675	2.35
94104	Q9Y5B6	PAX3 and PAX7 binding protein 1	PAXBP1	0	0	14753	0.00	13.85	0	0	46222	0.00	15.50	0.00	0.00	29981	0	14.87
158067	Q96MA6	Adenylate kinase 8	AK8	0	0	3707.9	0.00	11.86	0	0	26320	0.00	14.68	0.00	0.00	37086	0	15.18
222865	Q8N3G9	Transmembran e protein 130	TMEM130	0	0	13047	0.00	13.67	0	0	14532	0.00	13.83	0.00	0.00	40899	0	15.32
254048	Q6ZU65	Ubinuclein 2	UBN2	0	0	110170	0.00	16.75	0	0	49483	0.00	15.59	82050.00	0.00	244924	-16.32421	2.99
339766	A6NES4	Maestro heat like repeat family member 2A	MROH2A	0	0	49877	0.00	15.61	0	0	100740	0.00	16.62	0.00	0.00	10752	0	13.39
375033	Q5VY43	Platelet endothelial aggregation receptor 1	PEAR1	0	0	25394	0.00	14.63	0	0	92844	0.00	16.50	0.00	0.00	3840.2	0	11.91
494188	Q5MNV8	F-box protein 47	FBXO47	8705.9	0	132486	-13.09	3.93	0	0	16838	0.00	14.04	0.00	0.00	45521	0	15.47
728369	Q0WX57	Ubiquitin specific peptidase 17-like family member 24	USP17L24	5542.6	0	56992	-12.44	3.36	0	0	7222.2	0.00	12.82	8556.00	0.00	33126.2	-13.06272	3.87
1403640	Q80943	Replication protein E1	E1	0	0	12998	0.00	13.67	0	0	485160	0.00	18.89	3818.80	0.00	26530	-11.89890	2.80
105373377	A0A0J9YX94	Paraneoplastic antigen-like protein 6B	LOC1053733 77	14569	0	183835	-13.83	3.66	0	0	3533.1	0.00	11.79	0.00	0.00	91699	0	16.48

Table S3. The functions of protein-related with histone modification processes.

GO ID	GO Term	Term P Value	% Associated Genes
GO:0035405	Histone-threonine phosphorylation	6.52E-05	42.86
GO:0006348	Chromatin silencing at telomere	1.56E-05	30.77
GO:0098532	Histone H3-K27 trimethylation	2.18E-04	30.00
GO:0097692	Histone H3-K4 monomethylation	2.18E-04	30.00
GO:0098532	Histone H3-K27 trimethylation	2.18E-04	30.00
GO:0097692	Histone H3-K4 monomethylation	2.18E-04	30.00
GO:0042800	Histone methyltransferase activity (H3-K4 specific)	2.20E-06	27.78
GO:0070734	Histone H3-K27 methylation	2.27E-07	27.27
GO:0070734	Histone H3-K27 methylation	2.27E-07	27.27
GO:0080182	Histone H3-K4 trimethylation	2.96E-06	26.32
GO:0080182	Histone H3-K4 trimethylation	2.96E-06	26.32
GO:0080182	Histone H3-K4 trimethylation	2.96E-06	26.32
GO:0035173	Histone kinase activity	3.86E-05	25.00
GO:0043046	DNA methylation involved in gamete generation	3.91E-06	25.00
GO:0043968	Histone H2A acetylation	5.00E-05	23.53
GO:0018024	Histone-lysine N-methyltransferase activity	1.08E-11	23.40
GO:0018027	Peptidyl-lysine dimethylation	6.71E-07	23.08
GO:0051568	Histone H3-K4 methylation	2.51E-13	22.41
GO:0051568	Histone H3-K4 methylation	2.51E-13	22.41
GO:0031935	Regulation of chromatin silencing	6.37E-05	22.22
GO:0051571	Positive regulation of histone H3-K4 methylation	6.37E-05	22.22
GO:0031935	Regulation of chromatin silencing	6.37E-05	22.22
GO:0051571	Positive regulation of histone H3-K4 methylation	6.37E-05	22.22
GO:0046606	Negative regulation of centrosome cycle	6.36E-04	21.43
GO:0010826	Negative regulation of centrosome duplication	6.36E-04	21.43
GO:0033127	Regulation of histone phosphorylation	6.36E-04	21.43
GO:0090042	Tubulin deacetylation	6.36E-04	21.43
GO:0042054	Histone methyltransferase activity	9.09E-12	20.00
GO:1904837	Beta-catenin-TCF complex assembly	2.04E-06	19.35
GO:0070932	Histone H3 deacetylation	1.21E-04	19.05
GO:0034968	Histone lysine methylation	7.74E-21	18.85
GO:0034968	Histone lysine methylation	7.74E-21	18.85
GO:0031055	Chromatin remodeling at centromere	7.05E-09	18.75
GO:0006306	DNA methylation	7.79E-13	17.95
GO:0006305	DNA alkylation	7.79E-13	17.95
GO:0006306	DNA methylation	7.79E-13	17.95
GO:0006305	DNA alkylation	7.79E-13	17.95
GO:0061641	CENP-A containing chromatin organization	7.69E-08	17.78
GO:0034080	CENP-A containing nucleosome assembly	7.69E-08	17.78
GO:0016279	Protein-lysine N-methyltransferase activity	3.22E-10	17.46
GO:0016575	Histone deacetylation	1.80E-13	17.44
GO:0051569	Regulation of histone H3-K4 methylation	2.73E-05	17.24
GO:0061647	Histone H3-K9 modification	2.09E-08	16.67
GO:0016572	Histone phosphorylation	8.15E-07	16.67
GO:0018022	Peptidyl-lysine methylation	1.45E-19	16.67
GO:0018022	Peptidyl-lysine methylation	1.45E-19	16.67
GO:0006336	DNA replication-independent nucleosome assembly	2.48E-08	16.36
GO:0006476	Protein deacetylation	1.53E-14	16.19
GO:0140457	Protein demethylase activity	3.83E-05	16.13

GO:0032452	Histone demethylase activity	3.83E-05	16.13
GO:0070076	Histone lysine demethylation	3.83E-05	16.13
GO:0034724	DNA replication-independent nucleosome organization	2.92E-08	16.07
GO:0016571	Histone methylation	6.19E-20	16.00
GO:0016571	histone methylation	6.19E-20	16.00
GO:0031062	Positive regulation of histone methylation	1.13E-06	15.91
GO:0035065	Regulation of histone acetylation	4.02E-08	15.52
GO:0034508	Centromere complex assembly	4.02E-08	15.52
GO:0035065	Regulation of histone acetylation	4.02E-08	15.52
GO:0006342	Chromatin silencing	2.70E-10	15.19
GO:0006342	Chromatin silencing	2.70E-10	15.19
GO:0016577	Histone demethylation	5.24E-05	15.15
GO:0051567	Histone H3-K9 methylation	9.67E-06	15.00
GO:2000756	Regulation of peptidyl-lysine acetylation	1.03E-08	14.93
GO:2000756	Regulation of peptidyl-lysine acetylation	1.03E-08	14.93
GO:0033522	Histone H2A ubiquitination	3.34E-04	14.81
GO:0035601	Protein deacylation	8.47E-14	14.66
GO:0032451	Demethylase activity	1.12E-05	14.63
GO:0016574	Histone ubiquitination	2.09E-06	14.58
GO:0044728	DNA methylation or demethylation	2.80E-12	14.56
GO:0044728	DNA methylation or demethylation	2.80E-12	14.56
GO:0045814	Negative regulation of gene expression, epigenetic	1.83E-14	14.52
GO:1901983	Regulation of protein acetylation	2.59E-09	14.47
GO:1901983	Regulation of protein acetylation	2.59E-09	14.47
GO:0006482	Protein demethylation	7.02E-05	14.29
GO:0008214	Protein dealkylation	7.02E-05	14.29
GO:0004407	Histone deacetylase activity	7.02E-05	14.29
GO:0098732	Macromolecule deacylation	1.73E-13	14.05
GO:0033558	Protein deacetylase activity	8.07E-05	13.89
GO:0031060	Regulation of histone methylation	2.10E-08	13.89
GO:0043044	ATP-dependent chromatin remodeling	6.52E-12	13.76
GO:0018023	Peptidyl-lysine trimethylation	3.18E-06	13.73
GO:0018023	Peptidyl-lysine trimethylation	3.18E-06	13.73
GO:0043966	Histone H3 acetylation	1.28E-07	13.64
GO:0016569	Covalent chromatin modification	0.00E+00	13.24
GO:0016569	Covalent chromatin modification	0.00E+00	13.24
GO:0016570	Histone modification	0.00E+00	13.21
GO:0016570	Histone modification	0.00E+00	13.21
GO:0008276	Protein methyltransferase activity	1.46E-09	13.19
GO:0006333	Chromatin assembly or disassembly	4.46E-22	13.10
GO:0070317	Negative regulation of G0 to G1 transition	2.22E-05	13.04
GO:0031057	Negative regulation of histone modification	2.22E-05	13.04
GO:0031057	Negative regulation of histone modification	2.22E-05	13.04
GO:0031056	Regulation of histone modification	1.17E-15	12.96
GO:0031056	Regulation of histone modification	1.17E-15	12.96
GO:0031056	Regulation of histone modification	1.17E-15	12.96
GO:0010390	Histone monoubiquitination	5.76E-04	12.90
GO:0006479	Protein methylation	1.08E-17	12.90
GO:0008213	Protein alkylation	1.08E-17	12.90
GO:0006479	Protein methylation	1.08E-17	12.90
GO:0008213	Protein alkylation	1.08E-17	12.90
GO:0016573	Histone acetylation	2.19E-15	12.57
GO:0016573	Histone acetylation	2.19E-15	12.57

GO:0006338	Chromatin remodeling	7.70E-21	12.55
GO:1905268	Negative regulation of chromatin organization	1.45E-06	12.31
GO:1905268	Negative regulation of chromatin organization	1.45E-06	12.31
GO:0031497	Chromatin assembly	7.73E-18	12.25
GO:0031058	Positive regulation of histone modification	3.91E-09	12.12
GO:0070828	Heterochromatin organization	8.46E-08	12.05
GO:0043486	Histone exchange	3.94E-07	12.00
GO:0006475	Internal protein amino acid acetylation	6.41E-15	11.93
GO:0006475	Internal protein amino acid acetylation	6.41E-15	11.93
GO:0018394	Peptidyl-lysine acetylation	1.52E-15	11.89
GO:0018394	Peptidyl-lysine acetylation	1.52E-15	11.89
GO:0006325	Chromatin organization	0.00E+00	11.75
GO:0070316	Regulation of G0 to G1 transition	4.53E-05	11.54
GO:0040029	Regulation of gene expression, epigenetic	1.48E-16	11.54
GO:0043967	Histone H4 acetylation	2.58E-06	11.43
GO:1902275	Regulation of chromatin organization	1.25E-17	11.30
GO:1902275	Regulation of chromatin organization	1.25E-17	11.30
GO:1902275	Regulation of chromatin organization	1.25E-17	11.30
GO:0018205	Peptidyl-lysine modification	4.75E-32	11.27
GO:0018205	Peptidyl-lysine modification	4.75E-32	11.27
GO:0018205	Peptidyl-lysine modification	4.75E-32	11.27
GO:0018205	Peptidyl-lysine modification	4.75E-32	11.27
GO:0034401	Chromatin organization involved in regulation of transcription	1.64E-12	11.25
GO:0045023	G0 to G1 transition	5.62E-05	11.11
GO:0097549	Chromatin organization involved in negative regulation of transcription	3.95E-11	11.03
GO:0006473	Protein acetylation	2.55E-15	10.85
GO:0006473	Protein acetylation	2.55E-15	10.85
GO:0006334	Nucleosome assembly	5.40E-11	10.81
GO:0006323	DNA packaging	2.66E-18	10.73
GO:0043414	Macromolecule methylation	1.31E-22	10.67
GO:0043414	Macromolecule methylation	1.31E-22	10.67
GO:0043414	Macromolecule methylation	1.31E-22	10.67
GO:0034728	Nucleosome organization	6.23E-14	10.66
GO:0006304	DNA modification	4.99E-10	10.20
GO:0006304	DNA modification	4.99E-10	10.20
GO:0045652	Regulation of megakaryocyte differentiation	7.80E-06	9.88
GO:0045652	Regulation of megakaryocyte differentiation	7.80E-06	9.88
GO:0031507	Heterochromatin assembly	3.87E-05	9.46
GO:0031507	Heterochromatin assembly	3.87E-05	9.46
GO:1905269	Positive regulation of chromatin organization	1.94E-08	9.42
GO:0043543	Protein acylation	1.50E-13	8.98
GO:0043543	Protein acylation	1.50E-13	8.98
GO:0071103	DNA conformation change	5.94E-16	7.98
GO:0030219	Megakaryocyte differentiation	3.96E-05	7.92
GO:0030219	megakaryocyte differentiation	3.96E-05	7.92
GO:0051276	chromosome organization	0.00E+00	7.81
GO:0003714	transcription corepressor activity	1.16E-07	7.41
GO:0065004	protein-DNA complex assembly	5.30E-11	7.12
GO:0071824	protein-DNA complex subunit organization	3.87E-12	6.96
GO:2001252	positive regulation of chromosome organization	3.17E-07	6.83
GO:0016458	gene silencing	3.45E-09	6.67
GO:0003712	transcription coregulator activity	1.85E-09	5.02

GO:0002244	hematopoietic progenitor cell differentiation	5.29E-04	4.89
GO:0002244	hematopoietic progenitor cell differentiation	5.29E-04	4.89
GO:1901796	regulation of signal transduction by p53 class mediator	1.35E-04	4.87
GO:0003713	transcription coactivator activity	1.69E-05	4.86
GO:0045637	regulation of myeloid cell differentiation	1.12E-04	4.62
GO:0018193	peptidyl-amino acid modification	7.58E-19	4.44
GO:0018193	peptidyl-amino acid modification	7.58E-19	4.44
GO:0033044	regulation of chromosome organization	4.85E-05	4.42
GO:0072331	signal transduction by p53 class mediator	1.77E-04	4.13

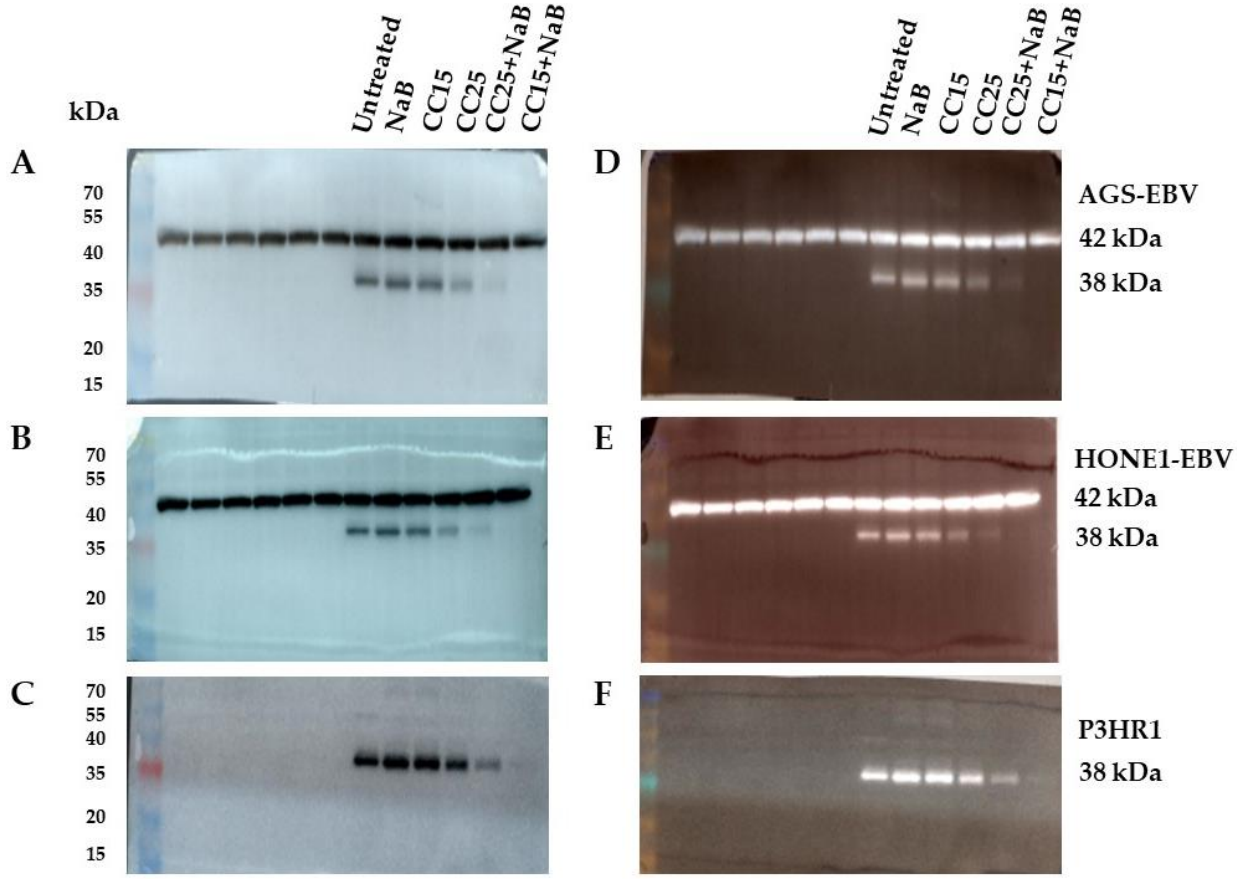

Figure S1. Andrographolide repressed EBV Zta expression. Cells were treated with andrographolide for 3 h, subsequently treated with NaB and further incubated for 48 h. The expression of Zta protein in cell lines were analyzed by western blotting. The inverted image of Zta protein expression of AGS-EBV (A), HONE1-EBV (B) and P3HR1 (C) cell lines. The uninvited image of Zta protein expression of AGS-EBV (D), HONE1-EBV (E) and P3HR1 (F) cell lines. Land 7: Untreated, Land 8: NaB, Land 9: CC_{15}, Land 10: CC_{25}, Land 11: a combination of andrographolide at CC_{25} and NaB, Land 12: a combination of andrographolide at CC_{25} and NaB.

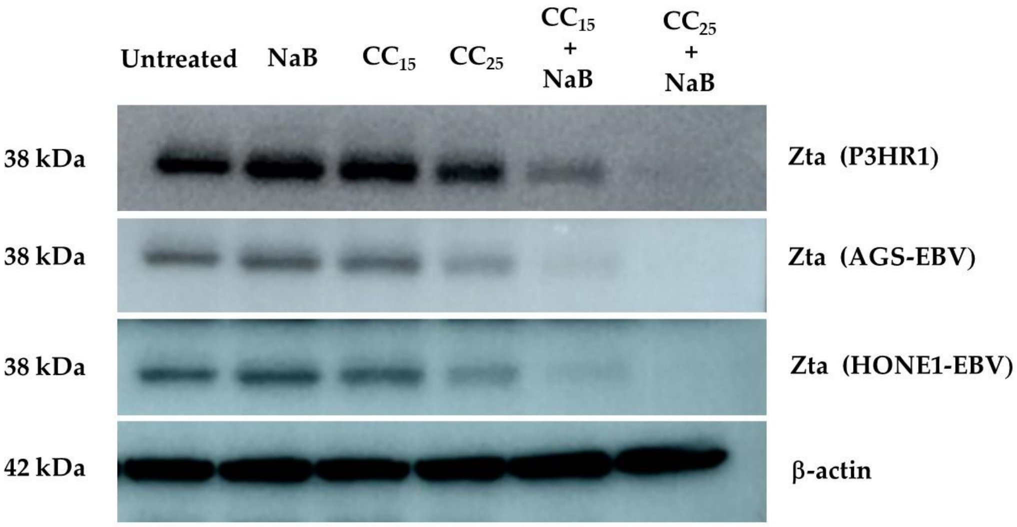

	Untreated		NaB		CC_{15}		CC_{25}		CC_{15} + NaB		CC_{25} + NaB	
Zta (P3HR1)/β-actin	0.7268	0.7091	1.2312	1.1830	0.6952	0.6238	0.6062	0.6703	0.4354	0.4251	0.1008	0.1089
Zta (AGS-EBV)/β-actin	0.3834	0.3628	0.5358	0.5601	0.3368	0.3247	0.1087	0.1315	0.0099	0.0100	0.0000	0.0000
Zta (HONE1-EBV)/β-actin	0.3649	0.3475	0.5433	0.5575	0.2320	0.2435	0.1111	0.1135	0.0173	0.0151	0.0000	0.0000

Figure S2. Western blot and densitometry reading/intensity ratio of Zta protein in cells treated with andrographolide. Cells were treated with andrographolide for 3 h, subsequently treated with NaB and further incubated for 48 h. The expression of Zta protein in cell lines were analyzed by western blotting.
